# The Effect of New Thiophene-Derived Aminophosphonic Derivatives on Growth of Terrestrial Plants: A Seedling Emergence and Growth Test

**DOI:** 10.3390/molecules21060694

**Published:** 2016-05-30

**Authors:** Jarosław Lewkowski, Zbigniew Malinowski, Agnieszka Matusiak, Marta Morawska, Diana Rogacz, Piotr Rychter

**Affiliations:** 1 Department of Organic Chemistry, Faculty of Chemistry, University of Łódź, Tamka 12, Łódź 91-403, Poland; zbigmal@uni.lodz.pl (Z.M.); agusk@poczta.fm (A.M.); martaem88@gmail.com (M.M.); 2 Faculty of Mathematics and Natural Science, Jan Długosz University in Częstochowa, 13/15 Armii Krajowej Av., Częstochowa 42-200, Poland; diana.rogacz@gmail.com

**Keywords:** thiophene-derived aminophosphonates, phytotoxicity, growth inhibition, environmental protection, ecotoxicology, OECD standard

## Abstract

The aim of this work was to synthesize selected thiophene-derived aminophosphonic systems and evaluate the phytotoxicity of newly obtained products according to the OECD 208 Guideline. Seven new thiophene-derived *N*-substituted dimethyl aminomethylphosphonic acid esters **2a**–**h** were synthesized by the addition of an appropriate phosphite to azomethine bond of starting Schiff bases **1a**–**h**, and NMR spectroscopic properties of aminophosphonates were investigated. These eight compounds were analyzed in regard to their phytotoxicity towards two plants, radish (*Raphanus sativus*) and oat (*Avena sativa*). On the basis of the obtained results, it was found that tested aminophosphonates **2a**–**h** showed an ecotoxicological impact against selected plants, albeit to various degrees.

## 1. Introduction

Nowadays, since production of food in agricultural areas is in the phase of intensive growth, the use of herbicides is absolutely necessary to manipulate or control undesirable vegetation of plants. Herbicides are used in landscapes throughout all over the world and are generally accepted as a moderately safe compounds. However, widespread use or overdosage of herbicides over long periods of time can result in residues in crops, soil, and land waters and, as a consequence, may lead to health and environmental risks [1,2,3,4]. Permanent applications of a given herbicide can result in resistance of a weed to the agent. Consequently, the weeds, while becoming resistant will not respond to the herbicide’s active properties [5,6]. With this respect, there is a necessity of designing new compounds with potential herbicidal properties.

Sulfur-containing compounds, which have been developed as agrochemicals for plant protection, are currently being reviewed from the standpoint of their use and biological properties in each field of fungicides, insecticides, or herbicides [7,8,9].

Sulfur-containing herbicides are successfully and widely used as a foliar (applied to weed foliage) and a soil-applied herbicide absorbed by the root or shoot of emerging seedlings.

Biodegradability, photodegradation, persistence, soil mobility, and the accumulation in living organisms of sulfur-containing herbicides are currently in extensive research phases [10,11,12,13,14].

Designing new sulfur-containing compounds with potential herbicidal activity in soil, their persistence in soil must be taken into consideration. On the one side, herbicides, which are applied to the soil, typically affect seed emergence or the growth of weed seedlings and must remain in the soil to be effective. Unfortunately, on the other side, some herbicides like thifensulfuron-methyl can persist in the environment for a long time, becoming harmful agents for the surrounding environment. Therefore, planning the synthesis, it is difficult to strike a balance between the activity of a compound and its biodegradability in soil [15].

Biological properties of thiophene-deriving aminophosphonates have been largely studied [16,17,18,19,20]. Scientists found several of them to be promising plant protection agents [16] or to have rather strong antimicrobial [17], cytotoxic [18], antifungal [19,20], or even antiviral [20] properties. Therefore, working on new variations of methods for synthesis of aminophosphonic systems, chemists include derivatives bearing thiophene moiety as examples [17,21,22,23,24,25,26]. It is to stress that all those methodologies are mostly based on the Kabachnik–Fields reaction [27,28,29].

Working on our large project aimed at the search of a new class of herbicidal agents, we feel obliged to perform tests of phytotoxicity of newly synthesized compounds, especially when these compounds are aminophosphonic derivatives, which are known generally to have moderate-to-strong phytotoxic action on higher plants [30,31,32,33].

Our previous investigation provided results demonstrating that aminophosphonic acids bearing furfuryl moiety (derivatives of *C*-furfurylphosphonoglycine) are compounds of moderate phytotoxicity being able to kill both monocotyledonous oat (*Avena sativa*) and dicotyledonous radish (*Raphanus sativus*) with an amount of 100–200 mg in 1 kg of soil [34].

The presented research is concerned with a screening test of a synthesized series of variously *N*-substituted dimethyl amino(2-thienyl)methylphosphonates (**2a**–**g**) and dibenzyl *N*-furfurylamino(2-thienyl)methylphosphonate (**2h**) with respect to their phytotoxic properties. Our assumption was that the potential hazard of thiophene-derived aminophosphonates is important enough in the light of their possible applications and therefore should be investigated.

Apart from that, we performed the preliminary evaluation of aminophosphonates **2a**–**h** as potential soil-applied herbicides for agricultural/horticultural purposes. The potential effects of herbicides strongly results from their mechanism of action and the way they are applied. Since some types of herbicides are non-selective, which means the chemicals kill all classes of plants, not only unwanted weeds, for the proposed experiment, but also two types of plants (mono- and dicotyledonous) have been chosen as experimental objects.

## 2. Results and Discussion

### 2.1. Chemistry

Schiff bases **1a**–**h** were synthesized following the published and commonly known procedure of simple mixing thiophene-2-carboxaldehyde with appropriate amine in methanol and stirring them at room temperature for 24 h [35]. This procedure produced imines **1a**–**h**, which were isolated and used for further conversions without any purification. ^1^H-NMR spectra were made only to verify the identity, based on the ^1^H-NMR diagnostic singlet of a proton of the azomethine group (–CH=N–) above 8 ppm [31,32].

**Scheme 1:**
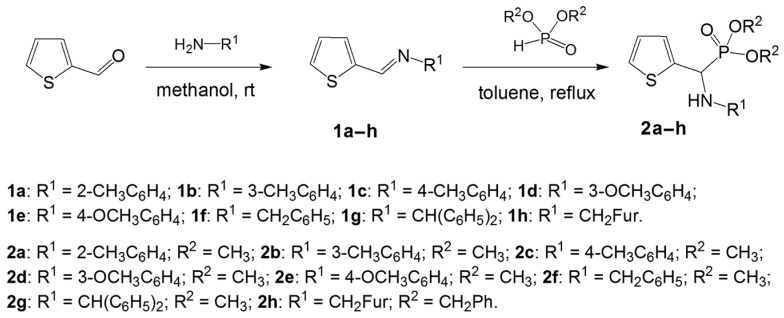
Synthesis of thiophene-derived aminophosphonates **2a**–**h**.

Aminophosphonates **2a**–**h** were synthesized basing on the aza-Pudovik reaction—a slightly modified procedure of a dimethyl or dibenzyl phosphite addition to the azomethine bond of corresponding Schiff bases **1a**–**h** in boiling toluene for 5 h. After the workup described in Section 3, the resulting aminophosphonates **2a**–**h** were obtained in 60%–70% yields (Scheme 1).

### 2.2. Evaluation of Phytotoxicity of Aminophosphonates ***2a–h***

The rate of growth and development of plants as well as their quality, including increased number and size of leaves and stems, is strongly dependent (as commonly known) on the ground composition. Since plants have a high amount of water, its level depends on the contents of water in its environment, so taking dry mass as a measure of plant growth seems to be more credible.

**Figure 1:**
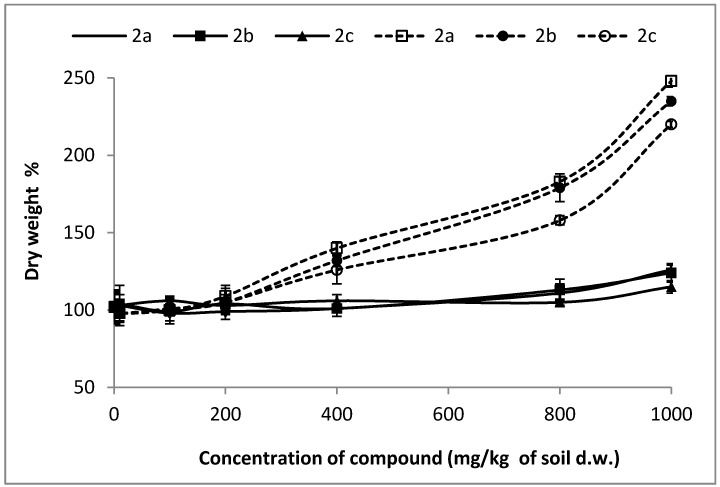
Changes of dry weight of treated plants expressed as percent to the value in untreated plants (control plants = 100% of dry weight). Solid lines represent changes of oat dry weight. Dotted lines represent changes of radish dry weight.

Water content is given as a percentage of the dry or fresh weight. To assess the phytotoxicity of the analyzed compounds, the emergence and weight (dry and green) of control plant seedlings, with the emergence and mass (dry and green) of plant seedlings growing on the soil with a given admixture of the examined substances **2a**–**h**, were determined and compared. Dry weight changes of tested plants are shown in Figure 1. The visual assessment of any damage of tested plants concerns mainly the degree of plant growth inhibition, which is evaluated by visible or other signs of necrosis of chlorosis. Necrosis is the symptom of the death of plant tissues or organs caused by infection. Leaf and/or stem deformation may also be observed, while chlorosis is an inhibition of chlorophyll biosynthesis, resulting in yellowing of tissue.

**Figure 2:**
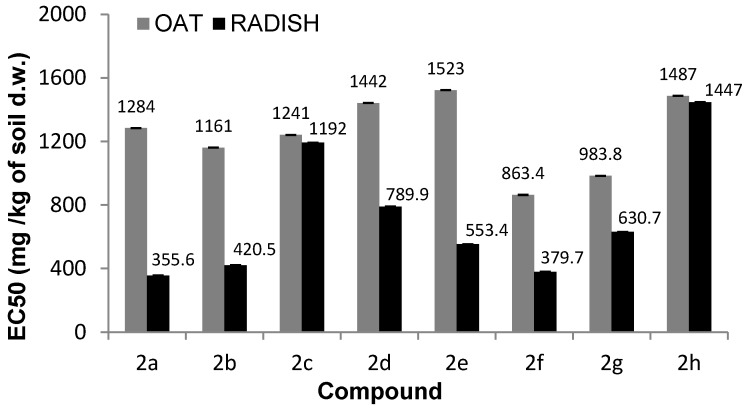
The EC_50_ values of fresh weight inhibition following exposure to tested compounds.

**Figure 3:**
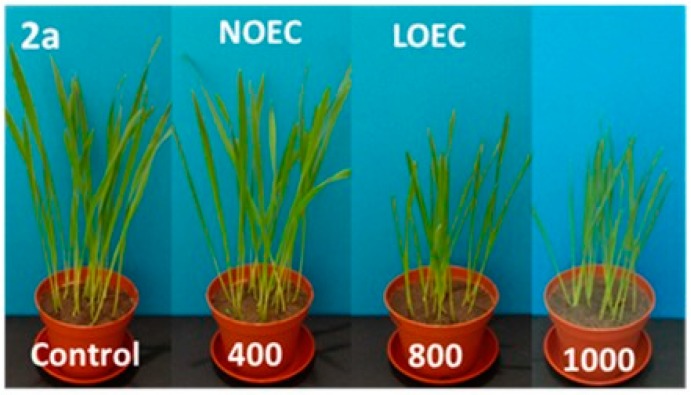

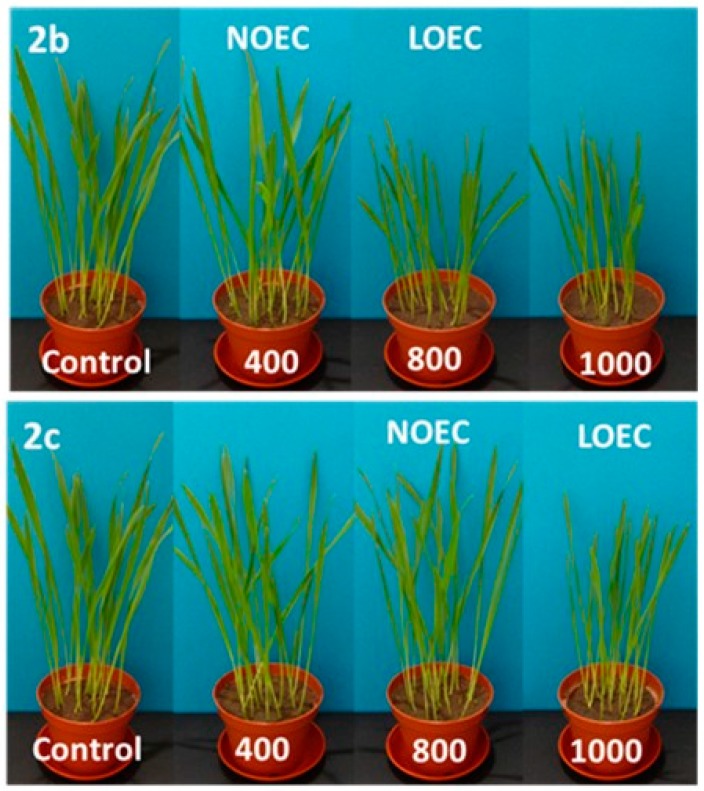
Digital photographs of oat treated with **2a**–**c** (concentration in mg/kg of soil dry weight) on the 14th day of growth.

**Figure 4:**
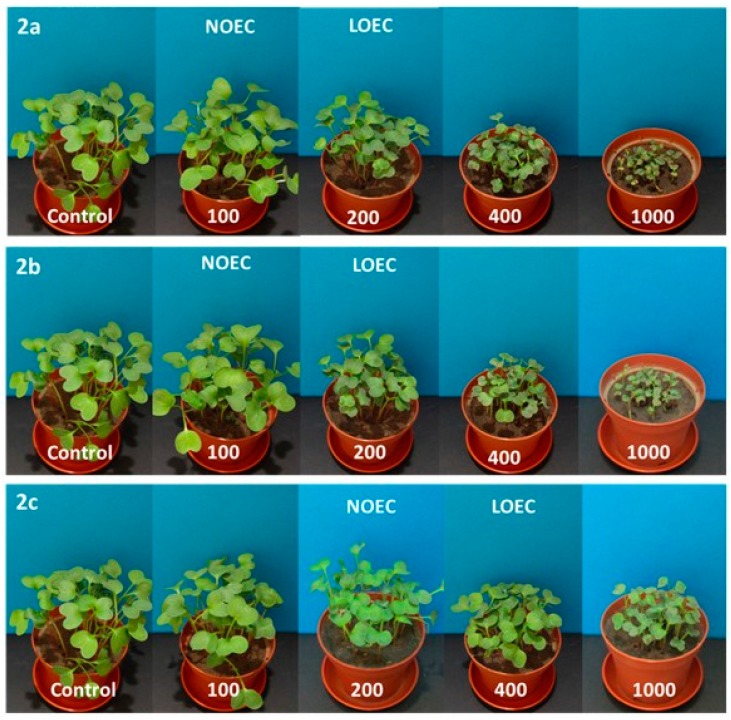
Digital photographs of radish treated with **2a**–**c** (concentration in mg/kg of soil dry weight) on the 14th day of growth.

Preliminary tests for aminophosphonates **2a**–**c** for both oat and radish have shown inhibitive effects for plants. The dicotyledonous radish was noticed as the more sensitive plant when compared to sprouts of oat. Comparing results of toxicity of aminophosphonates **2a**–**c** for both types of tested plants, it was noticed that Compounds **2a** and **2b** are more toxic as compared with **2c** (Table 1 and Table 2, Figure 1, Figure 2, Figure 3 and Figure 4).

*N*-methylphenyl aminophosphonates with *ortho* and *meta* substituted phenyl ring (**2a**, **2b**) have a significantly stronger influence on the inhibition of plant growth as compared with *N*-*para*-methylphenyl aminophosphonate (**2c**). In a case of oat, the values of NOEC and LOEC for Compounds **2a** and **2b** were 400 and 800 mg/kg of soil dry weight, respectively. These values for Compound **2c** were higher (NOEC and LOEC 800 and 1000 mg/kg d.w. of soil), indicating lower toxicity and subsequently higher tolerance of oat against this substance (Figure 3).

At the highest concentration (1000 mg), a *ca.* 40% decrease in fresh matter of oat sprouts for Compounds **2a** and **2b** when compared to control plants is observed (Table 1).

To make the description of the obtained results more readable and user-friendly, we only present selected tables and figures to the main text here (Table 1 and Table 2, Figure 1, Figure 2, Figure 3 and Figure 4).

Tested substances were much more toxic for the seedlings of radish, resulting in a decrease in NOEC and LOEC values. The first observed negative symptoms of tested **2a**–**c** compounds against radish were observed for the concentrations of 200 mg/kg of dry weight of soil (Samples **2a**, **2b**) and of 400 mg/kg of dry weight of soil for Compound **2c** (Figure 4). A very high drop of fresh weight of radish seedling was noticed for all compounds (**2a**–**c**), but aminophosphonates **2a** and **2b** were more toxic. At the concentration of 1000 mg/kg of dry weight of soil, a decrease in radish fresh weight, resulting from the action of Compounds **2a**–**c**, was 16%, 22%, and 55%, respectively. High values of dry matter at higher concentrations of the tested substances in pots correspond with a stronger inhibition of water uptake through the root system of plants (Figure 1). The increase in values of oat dry matter when compared to control plants is not as high as in the case of radish, which indicates better tolerance of oat roots against tested substances and moderately observed disturbances in the water uptake process.

The number of emerged seedlings at higher concentrations of tested compounds also decreased and was more visible for radish.

A plant growth test conducted for aminophosphonates bearing methoxy groups substituted in position *meta* (**2d**) and *para* (**2e**) in a phenyl ring have proved their toxic impact against both types of plants. Contrary to Compounds **2b**–**c** substituted with *meta*-methyl and *para*-methylphenyl groups, respectively, the examined *N-m-*methoxyphenyl derivative **2d** was less toxic for both radish and oat as compared with *N-p-*methoxyphenyl derivative **2e** (Tables S1 and S2, Figures S1–S3). The NOEC and LOEC values in the oat growth test for Compounds **2d** were much higher (800 and 1000 mg respectively) when compared to **2e** (100 and 200 mg respectively). The same values of NOEC and LOEC of Sample **2e** were revealed for radish. Again, as in the case of Compounds **2a**–**c**, the much more sensitive plant was radish. In comparison, the yield of the fresh matter of oat at the highest concentration (1000 mg) of **2d** and **2e** was 19% and 31% (respectively) lower compared to non-treated plants. The yield of the fresh weight of radish at the highest concentration did not exceed 29% (for **2d**) and 13% (for **2e**) of fresh matter of control plants. The high increase in dry matter values point out the dysfunction of roots (Figure S1).

Comparing the phytotoxicity results of aminophosphonates **2f**, **2g**, and **2h**, Compound **2h** was the most toxic agent for both plants. NOEC and LOEC values of Compound **2h** were the same for radish and oat—40 and 80 mg/kg of dry matter, respectively. Among Compounds **2f** and **2g**, the former had a higher negative impact on treated radish and oat. It was shown again that the more sensitive specimen of plant was radish. A very low yield of fresh matter for radish at the highest concentration (1000 mg) when compared to non-treated plants with simultaneously very high dry matter of plants indicates a strong inhibition of plant growth (both sprouts and roots) (Tables S3–S5, Figures S4–S8)

It is worth comparing the plant growth of Samples **2a**–**c** with **2f**, the non-aromatic amine derivative bearing the non-substituted phenyl ring. Sample **2f** is much more toxic against oat when compared to **2a**–**c**. In the case of radish, results are comparable to those obtained for Samples **2a**,**b** (the same values of NOEC and LOEC). Samples **2a** and **2b**, as well as *N*-benzyl derivative **2f**, in the highest applied concentration (1000 mg/kg), harmfully influenced radish seeds especially, while for oat, at the same concentration, Sample **2f** (without substituted phenyl ring with methyl group) caused almost total inhibition of sprout germination. As a result, contrary to dimethyl *N*-benzylamino(2-thienyl)methylphosphonate (**2f**), which kills both plants in a non-selective way, dimethyl *N*-(2-methylphenyl)amino(2-thienyl)methylphosphonate (**2a**) and dimethyl *N*-(3-methylphenyl)amino(2-thienyl)methylphosphonate (**2b**) were found to be selectively toxic towards radish, slightly harming oat.

## 3. Materials and Methods

### 3.1. Chemistry

All solvents (POCh, Gliwice, Poland) were routinely distilled and dried prior to use. Amines, dimethyl and dibenzyl phosphites, as well as 2-thiophenecarboxaldehyde (Aldrich, Poznań, Poland) were used as received. Melting points were measured on a MelTemp II apparatus (Bibby Scientific Limited, Staffordshire, UK) and were not corrected. NMR spectra were recorded on a Bruker Avance III 600 MHz (Billerica, MA, USA) operating at 600 MHz (^1^H-NMR), 150 MHz (^13^C-NMR), and 243 MHz (^31^P-NMR). TMS was used as the internal standard for ^1^H- and ^13^C-NMR, and phosphoric acid was used as the external standard for ^31^P-NMR. The following abbreviations were used for listing NMR signals: s—singlet, d—doublet, dd—doublet of doublets, ddd—doublet of doublet of doublets, and m—multiplet. Elemental analyses were carried out at the Centre for Molecular and Macromolecular Science of the Polish Academy of Science in Łódź, Poland.

#### General Procedures of Preparaing Amino(2-thienyl)methylphosphonates **2a**–**h**

2-Thiophenecarboxaldehyde (1.12 g, 10 mmol) was dissolved in methanol (15 mL), and a solution of an appropriate amine (10 mmol) in methanol (15 mL) was added. The obtained solution was stirred at room temperature for 24 h. Then, a small portion of anhydrous potassium carbonate was added, the mixture stirred for additional 15 min. Then, the inorganic salt was filtered off, and the filtrate was evaporated to achieve imines in quantitative yields, which were used for the further reaction without any purification.

Thus, the obtained imine (10 mmol) was dissolved in acetonitrile (15 mL), and an appropriate phosphite (10 mmol) in acetonitrile (15 mL) was added. The mixture was refluxed during the day and stirred at room temperature during the night. Total time of the reaction was 72 h. Then, the solvent was evaporated, and the crude product was isolated and purified using various procedures.

Dimethyl aminophosphonates **2a**–**g** were isolated as follows: crude products were dissolved in DCM (30 mL), and the solution was washed 5 times with saturated aqueous sodium hydrogen carbonate. After the usual workup, DCM was evaporated, and the product was purified by column chromatography on silica gel using ethyl acetate-hexane in a 4:1 ratio.

Product **2h** was chromatographed after evaporating acetonitrile, eluted using ethyl acetate—hexane in a 3:2 ratio.

*Dimethyl N-(2-methylphenyl)amino(2-thienyl)methylphosphonate* (**2a**): Yield = 83% (2.58 g) yellow oil. ^1^H-NMR (CDCl_3_, 600 MHz): δ 7.27–7.25 (m, H_5_^thioph^, 1H), 7.21–7.19 (m, H_3_^thioph^, 1H), 7.11 (d, ^3^*J*_HH_ = 7.4 Hz, *o*-C_6_H_4_, 1H), 7.07 (ddd, ^3^*J*^(1)^_HH_ = ^3^*J*^(2)^_HH_ = 7.4 Hz and ^4^*J*_HH_ = 1.7 Hz, *o*-C_6_H_4_, 1H), 7.01 (ddd, ^3^*J*_HH_ = 5.0 and 4.2 Hz and ^4^*J*_HH_ = 0.7 Hz, H_4_^thioph^, 1H), 6.74 (ddd, ^3^*J*^(1)^_HH_ = ^3^*J*^(2)^_HH_ = 7.4 Hz and ^4^*J*_HH_ = 1.0 Hz, *o*-C_6_H_4_, 1H), 6.65 (d, ^3^*J*_HH_ = 7.4 Hz, *o*-C_6_H_4_, 1H), 5.16 (d, ^2^*J*_PH_ = 23.7 Hz, CHP, 1H), 3.82 (d, ^3^*J*_PH_ = 10.6 Hz, POCH_3_, 3H), 3.67 (d, ^3^*J*_PH_ = 10.6 Hz, POCH_3_, 3H), 2.28 (s, CH_3_, 3H). ^13^C-NMR (CDCl_3_, 150 MHz): δ 143.98 (d, ^2^*J*_CP_ = 12.3 Hz, C^2^_thioph_), 139.81 (C_arom_), 130.41 (C_arom_), 127.19 (d, *J* = 2.5 Hz, C_thioph_), 127.03 (C_arom_), 126.19 (d, *J* = 7.2 Hz, C_thioph_), 125.43 (d, *J* = 3.4 Hz, C_thioph_), 123.39 (C_arom_), 118.86 (C_arom_), 111.48 (C_arom_), 54.17 (d, ^2^*J*_CP_ = 7.0 Hz, POC), 53.88 (d, ^2^*J*_CP_ = 7.3 Hz, POC), 51.84 (d, ^1^*J*_CP_ = 157.8 Hz, PC), 17.47 (ArC). ^31^P-NMR (243 MHz, CDCl_3_): δ 23.33. Elem. Anal. Calcd. for C_14_H_18_NO_3_PS: C, 54.01, H, 5.83, N, 4.50. Found: C, 54.18, H, 5.97, N, 4.59.

*Dimethyl N-(3-methylphenyl)amino(2-thienyl)methylphosphonate* (**2b**): Yield = 94% (2.92 g) yellow crystals, m.p.: 81–83 °C. ^1^H-NMR (CDCl_3_, 600 MHz): δ 7.26–7.25 (m, H_5_^thioph^, 1H), 7.21–7.20 (m, H_3_^thioph^, 1H), 7.07 (dd, ^3^*J*_HH_ = 7.2 and 7.0 Hz, *m*-C_6_H_4_, 1H), 7.01 (m, H_4_^thioph^, 1H), 6.62 (d, ^3^*J*_HH_ = 7.0 Hz, *m*-C_6_H_4_, 1H), 6.55 (s, *m*-C_6_H_4_, 1H), 6.52 (dd, ^3^*J*_HH_ = 7.2 Hz and ^4^*J*_HH_ = 1.9 Hz, *m*-C_6_H_4_, 1H), 5.11 (d, ^2^*J*_PH_ = 24.4 Hz, CHP, 1H), 3.82 (d, ^3^*J*_PH_ = 10.4 Hz, POCH_3_, 3H), 3.65 (d, ^3^*J*_PH_ = 10.8 Hz, POCH_3_, 3H), 2.28 (s, CH_3_, 3H). ^13^C-NMR (CDCl_3_, 150 MHz): δ 145.94 (d, ^2^*J*_CP_ = 13.1 Hz, C^2^_thioph_), 139.68 (C_arom_), 139.10 (C_arom_), 129.15 (C_arom_), 127.17 (d, *J* = 3.1 Hz, C_thioph_), 126.25 (d, *J* = 7.3 Hz, C_thioph_), 125.41 (d, *J* = 4.0 Hz, C_thioph_), 120.09 (C_arom_), 114.94 (C_arom_), 111.01 (C_arom_), 54.13 (d, ^2^*J*_CP_ = 6.7 Hz, PO*C*), 53.83 (d, ^2^*J*_CP_ = 7.4 Hz, PO*C*), 51.73 (d, ^1^*J*_CP_ = 158.4 Hz, P*C*), 21.54 (Ar*C*). ^31^P-NMR (243 MHz, CDCl_3_): δ 23.20. *Elem. Anal.* Calcd. for C_14_H_18_NO_3_PS: C, 54.01, H, 5.83, N, 4.50. Found: C, 54.17, H, 5.79, N, 4.51.

*Dimethyl N-(4-methylphenyl)amino(2-thienyl)methylphosphonate* (**2c**): Yield = 54% (1.68 g) yellow crystals, m.p.: 98–100 °C. ^1^H-NMR (CDCl_3_, 600 MHz): δ 7.25–7.24 (m, H_5_^thioph^, 1H), 7.20–7.19 (m, H_3_^thioph^, 1H), 7.05–6.98 (m, H_5_^thioph^, *p*-C_6_H_4_, 3H), 6.64 (part of AA’XX’ system, ^3^*J*_HH_ = 9.0 and ^4^*J*_HH_ = 1.2 and 1.1 Hz, *p*-C_6_H_4_, 2H), 5.09 (d, ^2^*J*_PH_ = 24.0 Hz, CHP, 1H), 3.82 (d, ^3^*J*_PH_ = 10.8 Hz, POCH_3_, 3H), 3.65 (d, ^3^*J*_PH_ = 10.8 Hz, POCH_3_, 3H), 2.24 (s, CH_3_, 3H). ^13^C-NMR (CDCl_3_, 150 MHz): δ 143.63 (d, ^2^*J*_CP_ = 13.4 Hz, C^2^_thioph_), 139.77 (C_arom_), 129.79 (C_arom_), 128.40 (C_arom_), 127.15 (d, *J* = 2.4 Hz, C_thioph_), 126.26 (d, *J* = 7.4 Hz, C_thioph_), 125.41 (d, *J* = 3.4 Hz, C_thioph_), 114.25 (C_arom_), 54.13 (d, ^2^*J*_CP_ = 7.1 Hz, PO*C*), 53.81 (d, ^2^*J*_CP_ = 7.1 Hz, PO*C*), 52.12 (d, ^1^*J*_CP_ = 157.9 Hz, P*C*), 20.40 (Ar*C*). ^31^P-NMR (243 MHz, CDCl_3_): δ 23.27. *Elem. Anal.* Calcd. for C_14_H_18_NO_3_PS: C, 54.01, H, 5.83, N, 4.50. Found: C, 54.13, H, 5.88, N, 4.63.

*Dimethyl N-(3-methoxyphenyl)amino(2-thienyl)methylphosphonate* (**2d**): Yield = 72% (2.35 g) yellow oil ^1^H-NMR (CDCl_3_, 600 MHz): δ 7.26–7.25 (m, H_5_^thioph^, 1H), 7.20–7.19 (m, H_3_^thioph^, 1H), 7.08 (dd, *J*_1_ = *J*_2_ = 7.8 Hz, *m*-C_6_H_4_, 1H), 7.01–6.99 (m, H_4_^thioph^, 1H), 6.35–6.34 (m, *m*-C_6_H_4_, 1H), 6.33–6.31 (m, *m*-C_6_H_4_, 1H), 6.28–6.26 (m, *m*-C_6_H_4_, 1H), 5.10 (d, ^2^*J*_PH_ = 24.0 Hz, CHP, 1H), 3.81 (d, ^3^*J*_PH_ = 10.8 Hz, POCH_3_, 3H), 3.74 (s, OCH_3_, 3H), 3.65 (d, ^3^*J*_PH_ = 10.8 Hz, POCH_3_, 3H). ^13^C-NMR (CDCl_3_, 150 MHz): δ 160.77 (C_arom_), 147.34 (d, ^2^*J*_CP_ = 13.5 Hz, C^2^_thioph_), 139.46 (C_arom_), 130.12 (C_arom_), 127.2 (d, *J* = 3.0 Hz, C_thioph_), 126.3 (d, *J* = 6.9 Hz, C_thioph_), 125.5 (d, *J* = 3.5 Hz, C_thioph_), 106.91 (C_arom_), 104.38 (C_arom_), 100.23 (C_arom_), 55.08 (ArO*C*), 54.12 (d, ^2^*J*_CP_ = 6.7 Hz, PO*C*), 53.90 (d, ^2^*J*_CP_ = 6.7 Hz, PO*C*), 51.69 (d, ^1^*J*_CP_ = 158.4 Hz, P*C*).^31^P-NMR (243 MHz, CDCl_3_: δ 23.06. *Elem. Anal.* Calcd. for C_14_H_18_NO_4_PS × ^1^/_5_ C_6_H_14_: C, 52.98, H, 6.08, N, 4.06. Found: C, 52.97, H, 5.96, N, 4.38.

*Dimethyl N-(4-methoxyphenyl)amino(2-thienyl)methylphosphonate* (**2e**): Yield = 80% (2.62 g) yellow oil. ^1^H-NMR (CDCl_3_, 600 MHz): δ 7.25–7.23 (m, H_5_^thioph^, 1H), 7.18–7.16 (m, H_3_^thioph^, 1H), 6.99–6.98 (m, H_4_^thioph^, 1H), 6.75 and 6.66 (AA’XX’ system, ^3^*J*_HH_ = 9.0 and ^4^*J*_HH_ = 1.2 and 1.1 Hz, *p*-C_6_H_4_, 4H), 4.94 (d, ^2^*J*_PH_ = 23.4 Hz, CHP, 1H), 4.54 (d, *J* = 8.4 Hz, NH, 1H), 3.81 (d, ^3^*J*_PH_ = 10.8 Hz, POCH_3_, 3H), 3.72 (s, OCH_3_, 3H), 3.64 (d, ^3^*J*_PH_ = 10.8 Hz, POCH_3_, 3H). ^13^C-NMR (CDCl_3_, 150 MHz): δ 153.26 (C_arom_), 139.91 (d, ^2^*J*_CP_ = 14.1 Hz, C^2^_thioph_), 139.73 (C_arom_), 127.1 (d, *J* = 2.8 Hz, C_thioph_), 126.3 (d, *J* = 6.9 Hz, C_thioph_), 125.4 (d, *J* = 4.0 Hz, C_thioph_), 115.69 (C_arom_), 114.83 (C_arom_), 55.63 (ArO*C*), 54.13 (d, ^2^*J*_CP_ = 6.7 Hz, PO*C*), 53.81 (d, ^2^*J*_CP_ = 6.7 Hz, PO*C*), 52.85 (d, ^1^*J*_CP_ = 158.4 Hz, P*C*). ^31^P-NMR (243 MHz, CDCl_3_): δ 23.33. *Elem. Anal.* Calcd. for C_14_H_18_NO_4_PS × ^1^/_6_ C_7_H_8_: C, 53.16, H, 5.69, N, 4.09. Found: C, 53.21, H, 5.78, N, 4.26.

*Dimethyl N-benzylamino(2-thienyl)methylphosphonate* (**2f**): Yield = 57% (1.77 g) yellow oil. ^1^H-NMR (CDCl_3_, 600 MHz): 7.26–7.20 (m, PhH, H_5_^thioph^, 5H), 7.18–7.15 (m, PhH, 1H), 7.02–7.01 (m, H_3_^thioph^, 1H), 6.93 (dd, ^3^*J*_HH_ = 4.8 and 3.6 Hz, H_4_^thioph^, 1H), 4.24 (d, ^2^*J*_PH_ = 23.8 Hz, CHP, 1H), 3.82 (d, ^2^*J*_HH_ = 13.8 Hz, CH_2_Ph, 1H), 3.68 (d, ^3^*J*_PH_ = 10.2 Hz, POCH_3_, 3H), 3.57 (d, ^2^*J*_HH_ = 13.8 Hz, CH_2_Ph, 1H), 3.55 (d, ^3^*J*_PH_ = 10.2 Hz, POCH_3_, 3H). ^13^C-NMR (CDCl_3_, 150 MHz): δ 139.30 (d, ^2^*J*_CP_ = 4.3 Hz, C^2^_thioph_), 138.99 (C_arom_), 128.47 (d, *J* = 7.5 Hz, C_thioph_), 128.38 (C_arom_), 128.23 (C_arom_), 127.28 (C_arom_), 126.97 (d, *J* = 3.5 Hz, C_thioph_), 125.68 (d, *J* = 3.3 Hz, C_thioph_), 54.74 (d, ^1^*J*_CP_ = 159.7 Hz, P*C*), 53.94 (d, ^2^*J*_CP_ = 6.8 Hz, PO*C*), 53.63 (d, ^2^*J*_CP_ = 6.8 Hz, PO*C*), 51.24 (d, ^3^*J*_CP_ = 15.6 Hz, PC*C*). ^31^P-NMR (243 MHz, CDCl_3_): δ 23.98. *Elem. Anal.* Calcd. for C_14_H_18_NO_3_PS: C, 54.01, H, 5.83, N, 4.50. Found: C, 53.77, H, 5.67, N, 4.46.

*Dimethyl N-benzhydrylamino(2-thienyl)methylphosphonate* (**2g**): Yield = 41% (1.59 g) yellow oil. ^1^H-NMR (CDCl_3_, 600 MHz): 7.44–7.34 (m, PhH, H_5_^thioph^, 6H), 7.32–7.22 (m, PhH, 5H), 7.07–7.06 (m, H_3_^thioph^, H_4_^thioph^, 2H), 4.91 (s, CH, 1H), 4.29 (d, ^2^*J*_PH_ = 22.8 Hz, CHP, 1H), 3.91 (d, ^3^*J*_PH_ = 10.8 Hz, POCH_3_, 3H), 3.64 (d, ^3^*J*_PH_ = 10.8 Hz, POCH_3_, 3H), 2.57–2.48 (bs, NH, 1H). ^13^C-NMR (CDCl_3_, 150 MHz): δ 139.36 (d, ^2^*J*_CP_ = 6.4 Hz, C^2^_thioph_), 139.73 (C_arom_), 128.75 (C_arom_), 128.56 (C_arom_), 127.89 (C_arom_), 127.17 (d, *J* = 7.9 Hz, C_thioph_), 127.07 (d, *J* = 2.3 Hz, C_thioph_), 125.72 (d, *J* = 3.4 Hz, C_thioph_), 63.81 (d, ^3^*J*_CP_ = 16.0 Hz, PC*C*), 54.12 (d, ^2^*J*_CP_ = 6.7 Hz, PO*C*), 53.53 (d, ^2^*J*_CP_ = 6.7 Hz, PO*C*), 53.19 (d, ^1^*J*_CP_ = 161.5 Hz, P*C*). ^31^P-NMR (243 MHz, CDCl_3_): δ 24.32. *Elem. Anal.* Calcd. for C_20_H_22_NO_3_PS × ^1^/_5_ C_7_H_8_: C, 63.33, H, 5.86, N, 3.45. Found: C, 63.54, H, 5.69, N, 3.75.

*Dibenzyl N-furfurylamino(2-thienyl)methylphosphonate* (**2h**): Yield = 62% (2.81 g) yellow oil. ^1^H-NMR (CDCl_3_, 600 MHz): δ 7.36–7.32 (m, PhH, 10H), 7.25–7.23 (m, H_5_^thioph^, H_5_^fur^, 1H), 7.11–7.09 (m, H_3_^thioph^, 1H), 7.03–7.02 (m, H_4_^thioph^, 1H), 6.33–6.31 (m, H_3_^fur^, 1H), 6.15–6.14 (m, H_4_^fur^, 1H), 5.10–5.02 (Part AB of ABX system, ^3^*J*_PH_ = 7.5 and 8.8 Hz, ^2^*J*_HH_ = 11.7 Hz, POCH_2_Ph, CHP, 2H), 5.00 (Part A of AMX system, ^3^*J*_PH_ = 7.2 and ^2^*J*_HH_ = 11.8 Hz, POCH_2_Ph, 1H), 4.90 (Part M of AMX system, ^3^*J*_PH_ = 8.2 and ^2^*J*_HH_ = 11.8 Hz, POCH_2_Ph, 1H), 4.43 (d, ^2^*J*_PH_ = 19.3 Hz, CHP, 1H), 3.92 and 3.69 (2d, ^2^*J*_HH_ = 14.6 Hz, CH_2_Fur, 3H). ^13^C-NMR (150 MHz, CDCl_3_): δ 152.60 (C_arom_), 142.12 (C_arom_), 138.67 (d, *J* = 4.8 Hz, C_arom_), 136.32 (d, ^3^*J*_CP_ = 6.0 Hz, C_arom_), 136.19 (d, ^3^*J*_CP_ = 6.3 Hz, C_arom_), 128.48 (d, *J* = 6.5 Hz, C_arom_), 128.34 (C_arom_), 128.28 (C_arom_), 128.00 (C_arom_), 127.84 (C_arom_), 127.36 (C_arom_), 127.30 (C_arom_), 126.92 (d, *J* = 2.4 Hz, C_arom_), 125.82 (d, *J* = 3.5 Hz, C_arom_), 110.14 (C_arom_), 107.92 (C_arom_), 68.58 and 68.18 (2d, ^2^*J*_CP_ = 40.6 Hz, PO*C*), 55.11 (d, ^1^*J*_CP_ = 159.8 Hz, P*C*), 43.72 (d, ^3^*J*_CP_ = 16.4 Hz, N*C*). ^31^P-NMR (243 MHz, CDCl_3_): δ 22.19. *Elem. Anal.* Calcd. for C_24_H_24_NO_4_PS × ^1^/_10_ CH_2_Cl_2_: C, 62.66, H, 5.28, .N, 3.03. Found: C, 62.77, H, 5.03, N, 3.05.

### 3.2. Plant Growth Test of New Synthesized Compounds

The plant growth test of thiophene-derived aminophosphonates **2a**–**h** was performed in laboratory conditions following the OECD 208 Guideline Terrestrial Plants Growth Test for oat (*Avena sativa*) as a monocotyledonous plant and radish (*Raphanus sativus* L. subvar. radicula Pers.), a dicotyledonous plant.

According to the mentioned OECD 208 standard, the plant growth test of Compounds **2a**–**h** was carried out in sandy soil having the following parameters: granulometric composition of soil—77% sand, 14% dust and loam, organic carbon content of approx. 1.2%, pH (KCl) equal to 6.3.

Tests were carried out in polypropylene pots (diameter of 90 mm and capacity of 300 cm^3^), which were filled with the control soil or with the soil mixed with the tested Compounds **2a**–**h** added at a specific concentration. Twenty identical seeds of each of the selected plant species were sown into the soil. Seeds originated from the same source. Plants were grown for 14 days under controlled conditions: a constant humidity content at the level required for the plants (70% field water capacity), light intensity (7000 lux), and temperature (20 ± 2 °C) were maintained. Then, seedlings were counted, and the dry as well as fresh weight of the plants was determined. The parts of plants above the soil surface were analyzed.

The performed plant growth was evaluated using a preliminary test; subsequently, the final test was conducted afterward based on the obtained screening results. Preliminary tests were performed to determine the range of concentrations of compounds affecting the soil quality; therefore, the used concentrations of Samples **2a**–**h** were as follows: 0 mg (control), 1 mg, 10 mg, 100 mg, and 1000 mg/kg of soil dry weight.

As already mentioned, based on the obtained results from preliminary test, the final, more precise test to find values of the no observed effect concentration (NOEC) and the lowest observed effect concentration (LOEC) of the compounds under study **2a**–**h** was performed.

The evaluation of phytotoxicity of the studied aminophosphonates **2a**–**h** at applied concentrations was made by comparing the germination and (dry and fresh) weight of control plant sprouts (seedlings) with germination and of (dry and fresh) plants sprouts grown in the soil with an admixture of given amounts of the tested compounds.

The dry weights of tested plants were measured after drying at 75 °C until the constant weight.

The visual evaluation of phytotoxicity of aminophosphonates **2a**–**h** at applied concentrations was performed by digital photography. Obtained pictures were analyzed in terms of any type of damage to tested plants, such as their growth inhibition, chlorosis, and necrosis. Tests were carried out three times for each sample.

The significance of the obtained results was evaluated using the analysis of variance (ANOVA). The least significant difference (LSD) values at a confidence level of 95% were computed using the Tukey test.

## 4. Conclusions

To conclude, the toxicity of *N*-methylphenyl aminophosphonates **2a**–**c** was diagnosed to be promising as a potential soil-applied herbicide, especially for their selectivity—they are evidently toxic for dicotyledonous radish and not so harmful for monocotyledonous oat. Moreover, their herbicidal efficiency is stronger for the *N*-methylphenyl aminophosphonates substituted with methyl group at position *ortho* (**2a**) and *meta* (**2b**) in the phenyl ring.

Dimethyl *N*-benzylamino(2-thienyl)methylphosphonate (**2f**) exhibited non-selective, harmful effects against both tested plants.

Among *N*-methoxyphenyl aminophosphonates **2d** and **2e**, the latter showed an inhibitive effect especially against dicotyldenous plants. Its impact, however, was significantly weaker than impact of **2a**,**b**. This is to point out that the sole structural difference between *N*-methoxyphenyl aminophosphonates **2d**,**e** and *N*-methylphenyl aminophosphonates **2a**–**c** is the substituent of the phenyl moiety, *i.e.*, the methoxy and methyl group, respectively, which indicates that the type and position of those groups in the phenyl ring play a key role in the toxicity effect against tested plants.

Among all tested samples, dibenzyl *N*-furfurylamino(2-thienyl)methylphosphonate (**2h**) was found to be the most toxic for radish and oat but without significant selectivity. The investigation of Compounds **2a**,**b**, **2f**, and **2h**, such as their potential applications and mechanistic approaches, will be continued.

Some thiophene-derived aminophosphonic derivatives were found to have various biological properties (*vide supra*); therefore, important microbiological, herbicidal, and cytotoxic properties of the studied aminophosphonic systems bearing thiophene moiety **2a**–**h** cannot be excluded. If so, the synthesis of such compounds for potential agrochemical or pharmacological application must be taken into consideration from the environmental protection point of view. Our results call attention to the necessity of further phytotoxicological investigation of any new synthesized aminophosphonic derivatives bearing thiophene moiety.

## Figures and Tables

**Table 1:** Average changes (mean of three replicates) in basic parameters of the plant growth test for oat (Avena sativa) treated with **2a**–**c**. Least significant difference for samples (LSDS) and concentration (LSDC) is given for each tested parameter. %F.M. refers to plant biomass (fresh weight) expressed as percent of untreated control.

Sample Concentration in Soil (mg/kg of Soil Dry Matter)	Emerged Seedlings Number	% of Germination	Fresh Matter (g/pot)	% F.M.
0	20	100	2.780	100
1	19	97	2.682	96
10	19	95	2.654	95
100	19	97	2.919	105
200	19	95	2.644	95
400	19	95	2.715	98
800	18	88	2.100	76
1000	18	90	1.847	66
0	20	100	2.780	100
1	19	95	2.740	99
10	19	97	2.663	96
100	19	97	2.729	98
200	19	97	2.651	95
400	20	98	2.661	96
800	18	92	2.021	73
1000	18	92	1.659	60
0	20	100	2.780	100
1	19	97	2.759	99
10	20	98	2.704	97
100	20	98	2.801	101
200	19	97	2.862	103
400	20	98	2.762	99
800	19	95	2.643	95
1000	19	95	2.253	81
	LSDS = 1		LSDS = 0.401	
	LSDC = 1		LSDC = 0.245	

All the other results, to avoid confusion concerned with a large amount of data, have been placed in Supplementary Materials. To describe results, moreover, the demonstrated values of no observed effect concentration (NOEC) and lowest observed effect concentration (LOEC), changes in the fresh mass of plants were expressed as % inhibition compared to control and presented as EC_50_ (Figure 2). Effective concentration EC_50_ was calculated using GraphPad Prism software (Version 7, GraphPad Software, Inc., La Jolla, CA 92037, USA).

**Table 2:** Average changes (mean of three replicates) in basic parameters of the plant growth test for radish (*Raphanus sativus*) treated with **2a**–**c**.

Sample Concentration in Soil (mg/kg of Soil Dry Matter)	Emerged Seedlings Number	% of Germination	Fresh Matter (g/pot)	% F.M.
0	20	100	4.951	100
1	20	100	4.908	99
10	19	97	4.831	98
100	19	98	4.728	95
200	18	93	3.928	79
400	16	83	1.752	35
800	18	90	1.136	23
1000	15	75	0.772	16
0	20	100	4.951	100
1	20	100	4.803	97
10	19	95	4.843	98
100	19	97	4.728	95
200	19	97	3.928	79
400	17	86	2.142	43
800	17	88	1.546	31
1000	15	78	1.106	22
0	20	100	4.951	100
1	19	98	4.914	99
10	19	98	4.798	97
100	19	98	4.873	98
200	19	97	4.787	97
400	18	93	3.934	79
800	18	93	3.328	67
1000	17	86	2.733	55
	LSD_S_ = 1		LSD_S_ = 0.435	
	LSD_C_ = 1		LSD_C_ = 0.267	

Least significant difference for samples (LSD_S_) and concentration (LSD_C_) is given for each tested parameter. %F.M. refer to plant biomass (fresh weight) expressed as percent of untreated control.

## Supplementary Materials

Supplementary materials can be accessed at: http://www.mdpi.com/1420-3049/21/6/694/s1.
