# Supplementary material for: The Effect of New Thiophene-Derived Aminophosphonic Derivatives on Growth of Terrestrial Plants: A Seedling Emergence and Growth Test
Source: Molecules. 2016 May 30;21(6):694. doi: 10.3390/molecules21060694 (PMC6272962; doi:10.3390/molecules21060694)
Supplement: Supplementary file 1 [file molecules-21-00694-s001.pdf]

Supplementary Materials: The Effect of New Thiophene-Derived Aminophosphonic Derivatives on Growth of Terrestrial Plants: A Seedling Emergence and Growth Test

Jarosław Lewkowski, Zbigniew Malinowski, Agnieszka Matusiak, Marta Morawska, Diana Rogacz and Piotr Rychter

Figures S1, S4 and S7 (changes of dry weight of treated plants), Figures S2, S3, S5–S7 (photographs of studied plants) and Tables S1–S5 page S1
Figure S9–S32. ^{1}H, ^{13}C and ^{31}P-NMR spectra of Compounds **2a**–**h** page S8–S31

Table S1. Average changes (mean of three replicates) in basic parameters of the plant growth test for oat *(Avena sativa)* treated with **2d** and **2e**. Least significant difference for samples (LSD_S) and concentration (LSD_C) is given for each tested parameter. % F.M. refer to plant biomass (fresh weight) expressed as percent of untreated control.

Sample Concentration in Soil (mg/kg of Soil Dry Matter)	Emerged Seedlings Number	% of Germination	Fresh Matter (g/pot)	% F.M.
**0**	20	100	2.780	100
**1**	19	97	2.688	97
**10**	19	97	2.663	96
**100**	20	98	2.819	101
**200**	19	97	2.675	96
**400**	19	95	2.691	97
**800**	19	95	2.566	92
**1000**	18	90	2.249	81
**0**	20	100	2.780	100
**1**	19	95	2.682	96
**10**	19	97	2.645	95
**100**	19	97	2.724	98
**200**	20	98	2.447	88
**400**	19	95	2.304	83
**800**	18	92	2.026	73
**1000**	18	88	1.921	69
	$LSD_S = 1$ $LSD_C = 1$		$LSD_S = 0.3$ $LSD_C = 0.15$	

Table S2. Average changes (mean of three replicates) in basic parameters of the plant growth test for radish *(Raphanus sativus)* treated with **2d** and **2e**. Least significant difference for samples (LSD_S) and concentration (LSD_C) is given for each tested parameter. % F.M. refer to plant biomass (fresh weight) expressed as percent of untreated control.

Sample Concentration in Soil (mg/kg of Soil Dry Matter)	Emerged Seedlings Number	% of Germination	Fresh Matter (g/pot)	% F.M.
**0**	20	100	4.951	100
**1**	19	98	4.850	98
**10**	19	98	4.811	97
**100**	20	100	4.735	96
**200**	19	95	4.934	100
**400**	19	95	4.149	84
**800**	17	86	2.719	55
**1000**	17	86	1.440	29
**0**	20	100	4.951	100
**1**	20	100	4.841	98

Table S2. *Cont.*

Sample Concentration in Soil (mg/kg of Soil Dry Matter)	Emerged Seedlings Number	% of Germination	Fresh Matter (g/pot)	% F.M.
**10**	19	95	4.745	96
**100**	19	97	4.842	98
**200**	19	97	4.035	81
**400**	17	86	3.748	76
**800**	17	85	1.436	29
**1000**	15	76	0.662	13
	LSDs = 2		LSDs = 0.492	
	LSDc = 1		LSDc = 0.246	

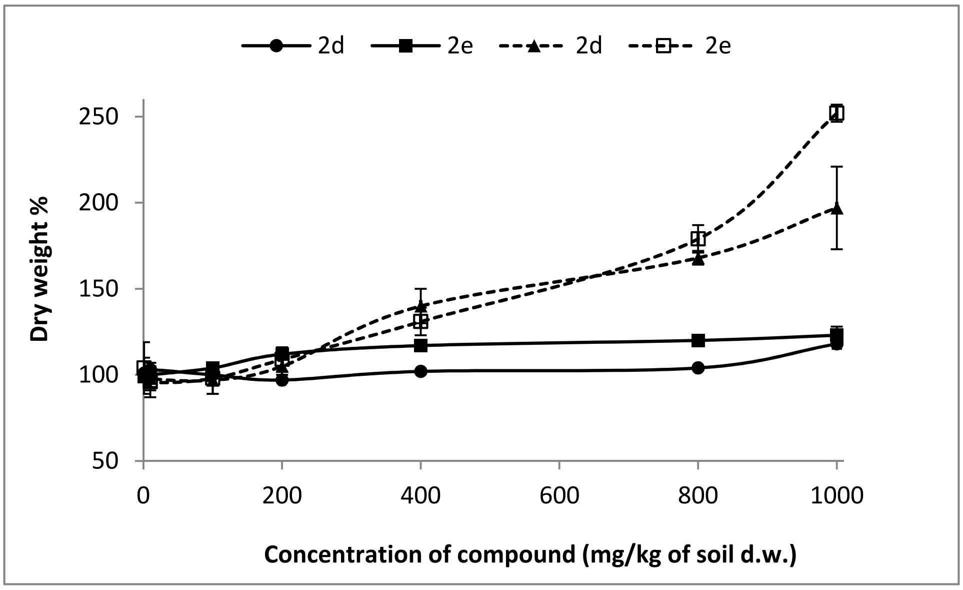

Figure S1. Changes of dry weight of treated plants with Compounds **2d** and **2e** expressed as percent to the value in untreated plants (control plants = 100% of dry weight). Solid lines represent changes of oat dry weight. Dotted lines represent changes of radish dry weight.

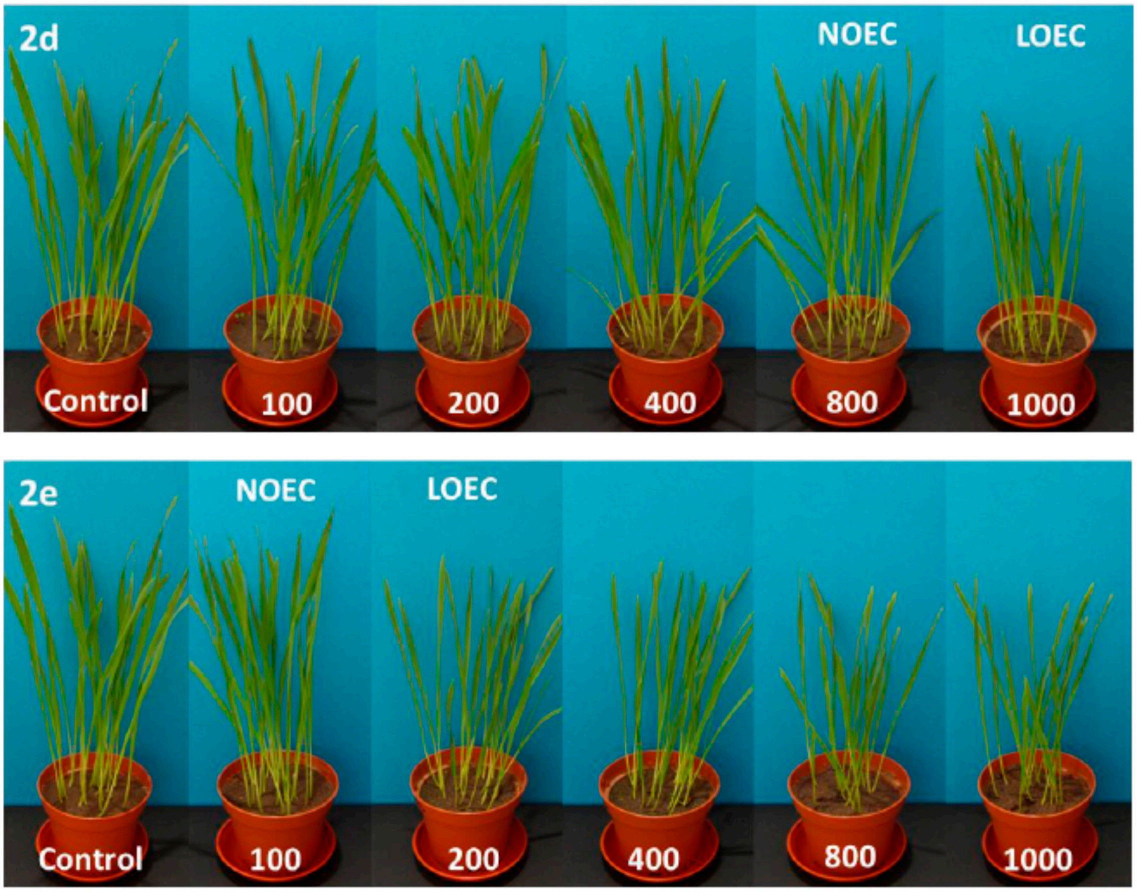

Figure S2. Digital photographs of oat treated with **2d** and **2e** (concentration in mg/kg of soil dry weight) on the 14th day of growth.

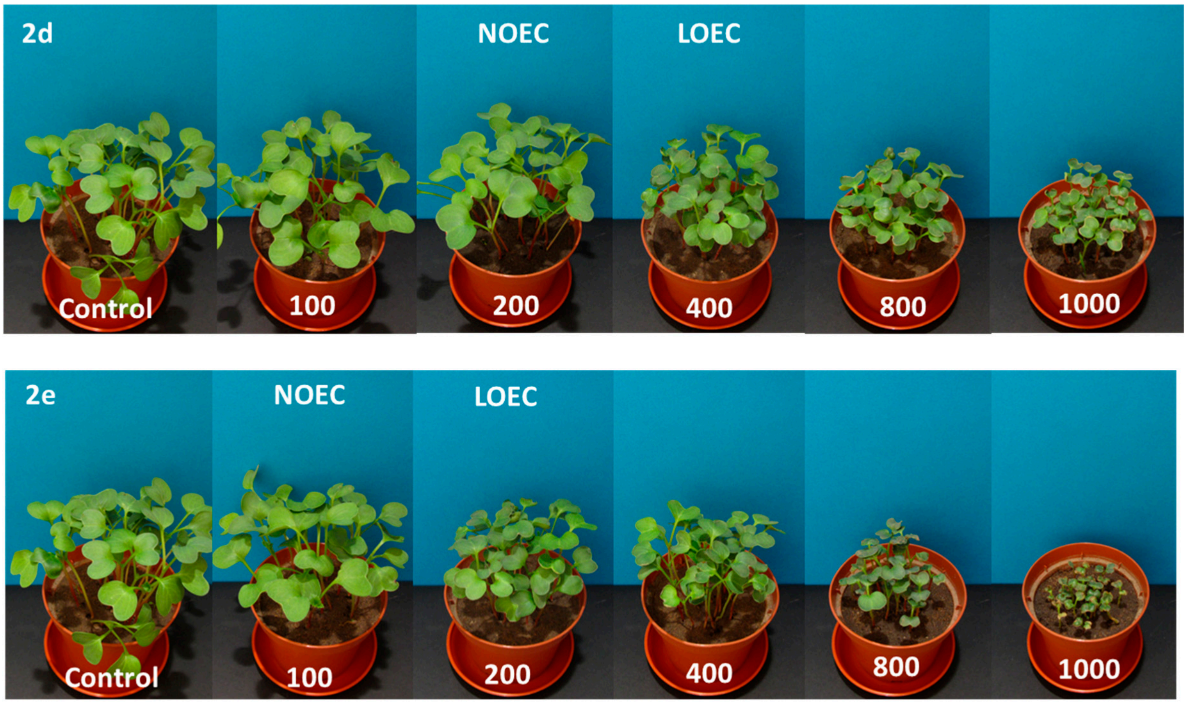

Figure S3. Digital photographs of radish treated with **2d** and **2e** (concentration in mg/kg of soil dry weight) on the 14th day of growth.

Table S3. Average changes (mean of three replicates) in basic parameters of the plant growth test for oat *(Avena sativa)* treated with **2f** and **2g**. Least significant difference for samples (LSDs) and concentration (LSDc) is given for each tested parameter. % F.M. refer to plant biomass (fresh weight) expressed as percent of untreated control.

Sample Concentration in Soil (mg/kg of Soil Dry Matter)	Emerged Seedlings Number	% of Germination	Fresh Matter (g/pot)	% F.M.
**0**	20	100	2.807	100
**1**	19	97	2.761	98
**10**	19	97	2.729	97
**100**	20	98	2.800	100
**200**	19	97	2.769	99
**400**	19	95	2.434	87
**800**	17	87	1.949	69
**1000**	14	70	0.483	17
**0**	20	100	2.807	100
**1**	20	98	2.752	98
**10**	19	97	2.771	99
**100**	20	98	2.887	103
**200**	19	97	2.784	99
**400**	20	98	2.728	97
**800**	18	90	2.340	83
**1000**	17	87	1.315	47
	LSDs = 2		LSDs = 0.205	
	LSDc = 1		LSDc = 0.102	

Table S4. Average changes (mean of three replicates) in basic parameters of the plant growth test for radish *(Raphanus sativus)* treated with **2f** and **2g**. Least significant difference for samples (LSDs) and concentration (LSDc) is given for each tested parameter. % F.M. refer to plant biomass (fresh weight) expressed as percent of untreated control.

Sample Concentration in Soil (mg/kg of Soil Dry Matter)	Emerged Seedlings Number	% of Germination	Fresh Matter (g/pot)	% F.M.
**0**	20	100	4.821	100
**1**	20	100	4.793	99
**10**	20	100	4.612	96
**100**	19	98	4.720	98
**200**	17	88	4.147	86
**400**	17	85	2.070	43
**800**	14	71	0.860	18
**1000**	12	61	0.394	8
**0**	20	100	4.821	100
**1**	20	100	4.765	99
**10**	19	95	4.700	97
**100**	19	95	4.741	98
**200**	19	98	4.543	94
**400**	17	85	3.410	71
**800**	15	76	1.920	40
**1000**	14	73	1.231	26
	LSDs = 2		LSDs = 0.368	
	LSDc = 1		LSDc = 0.184	

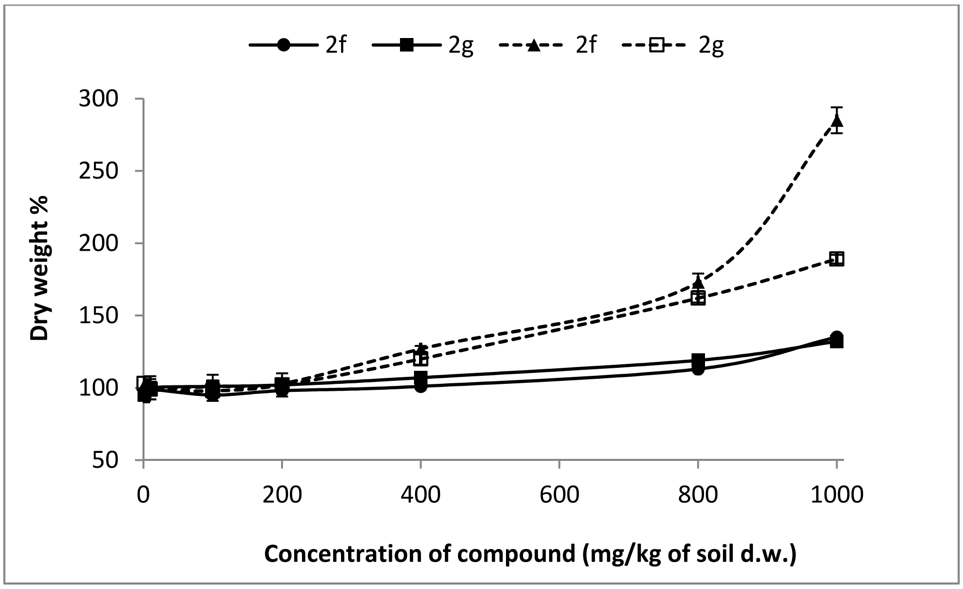

Figure S4. Changes of dry weight of treated plants with Compounds **2f** and **2g** expressed as percent to the value in untreated plants (control plants = 100% of dry weight). Solid lines represent changes of oat dry weight. Dotted lines represent changes of radish dry weight.

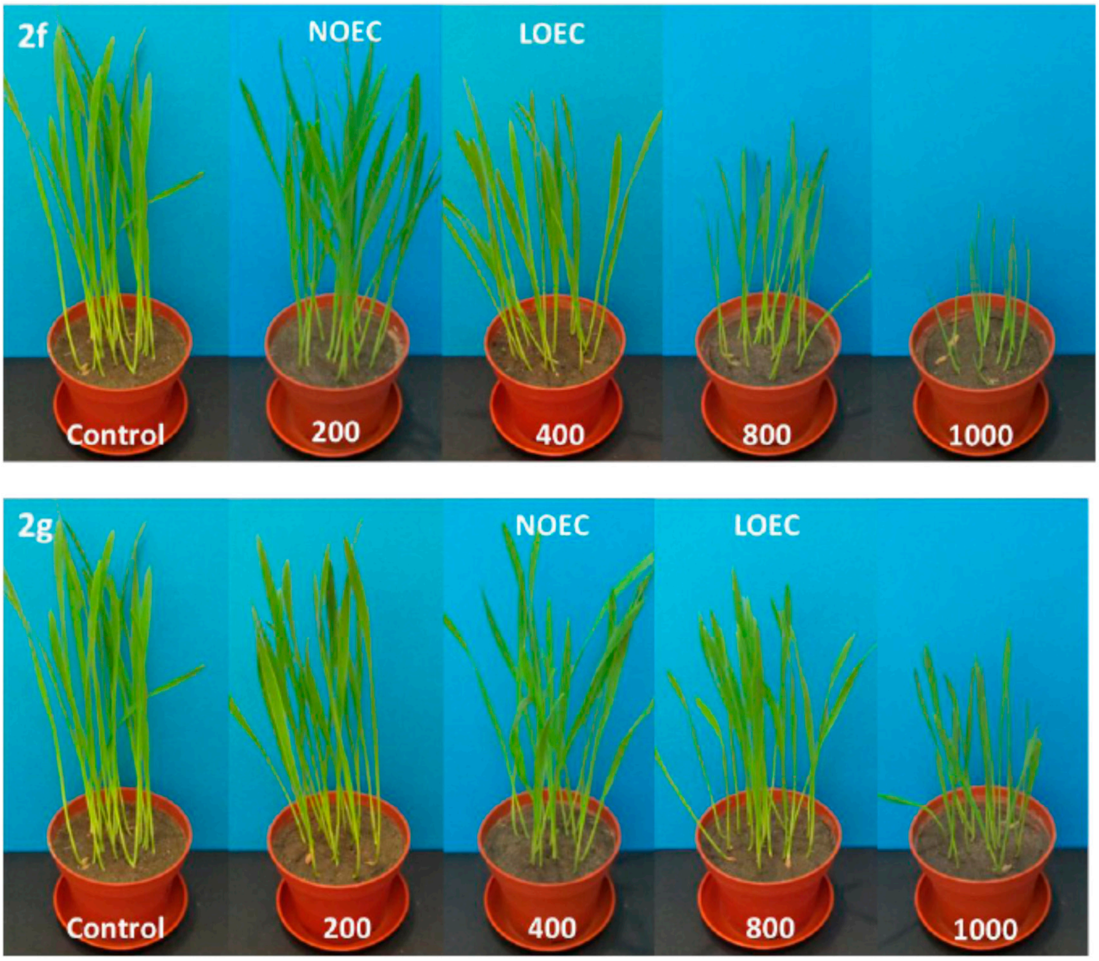

Figure S5. Digital photographs of oat treated with **2f** and **2g** (concentration in mg/kg of soil dry weight) on the 14th day of growth.

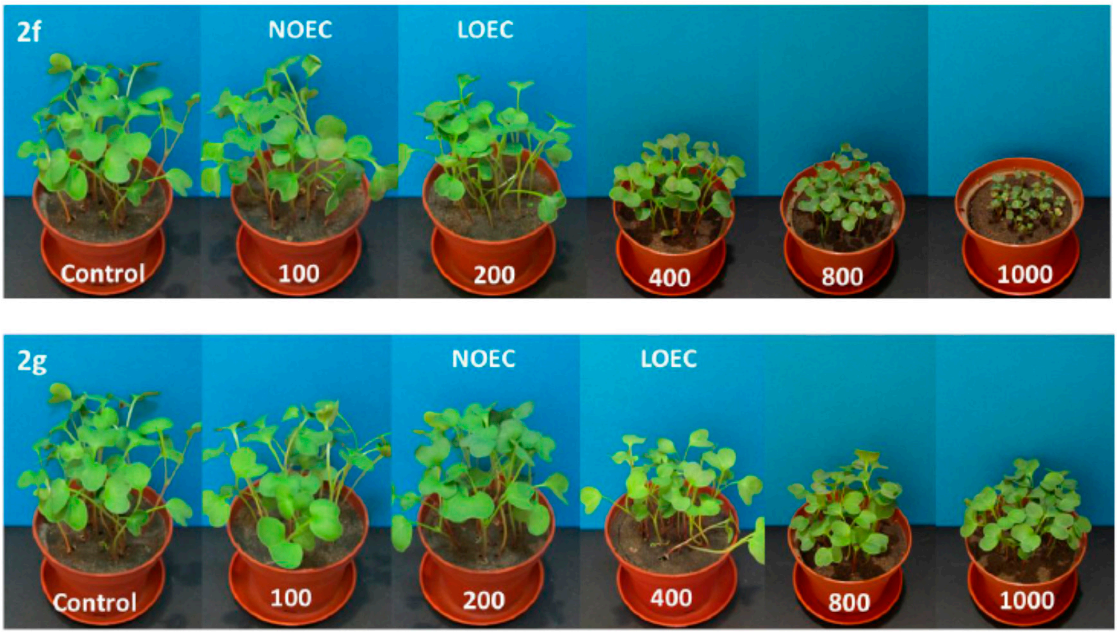

Figure S6. Digital photographs of radish treated with **2f** and **2g** (concentration in mg/kg of soil dry weight) on the 14th day of growth.

Table S5. Average changes (mean of three replicates) in basic parameters of the plant growth test for oat and radish treated with **2h.** Least significant difference for concentration (LSD) is given for each tested parameter. %F.M. refer to plant biomass (fresh weight) expressed as percent of untreated control.

Sample Concentration in Soil (mg/kg of Soil Dry Matter)	Emerged Seedlings Number	% of Germination	Fresh Matter (g/pot)	% F.M.
		OAT		
**0**	20	100	2.772	100
**1**	20	98	2.742	99
**10**	20	98	2.714	98
**20**	19	97	2.767	100
**40**	19	97	2.731	99
**80**	19	97	2.359	85
**100**	19	95	2.186	79
**1000**	17	87	1.730	62
	LSD = 1		LSD = 0.126	
		RADISH		
**0**	20	100	4.799	100
**1**	19	98	5.112	107
**10**	19	97	4.686	98
**20**	19	97	4.711	98
**40**	19	97	4.496	94
**80**	18	90	4.226	88
**100**	16	81	3.948	82
**1000**	13	66	2.867	60
	LSD = 1		LSD = 0.181	

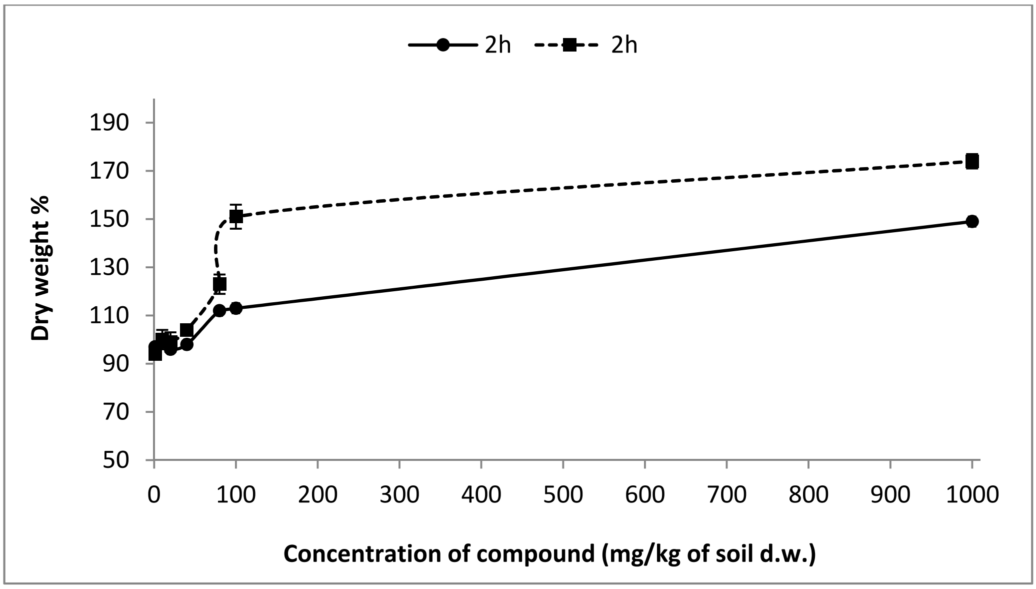

Figure S7. Changes of dry weight of treated plants with Compound **2h** expressed as percent to the value in untreated plants (control plants = 100% of dry weight). Solid lines represent changes of oat dry weight. Dotted lines represent changes of radish dry weight.

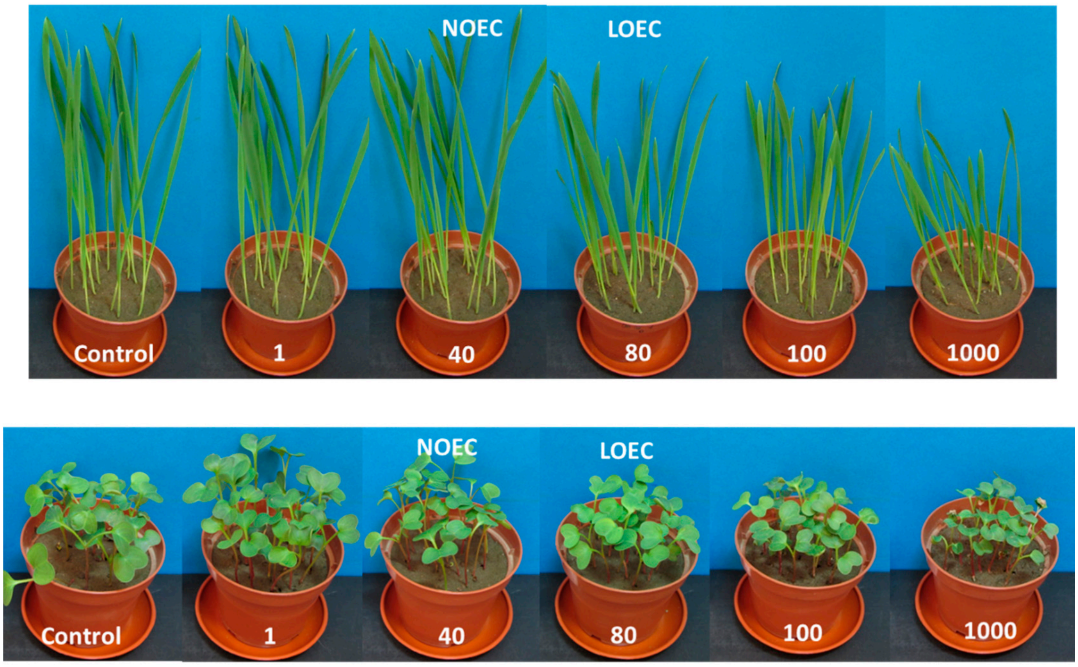

Figure S8. Digital photographs of oat and radish treated with **2h** (concentration in mg/kg of soil dry weight) on the 14th day of growth.

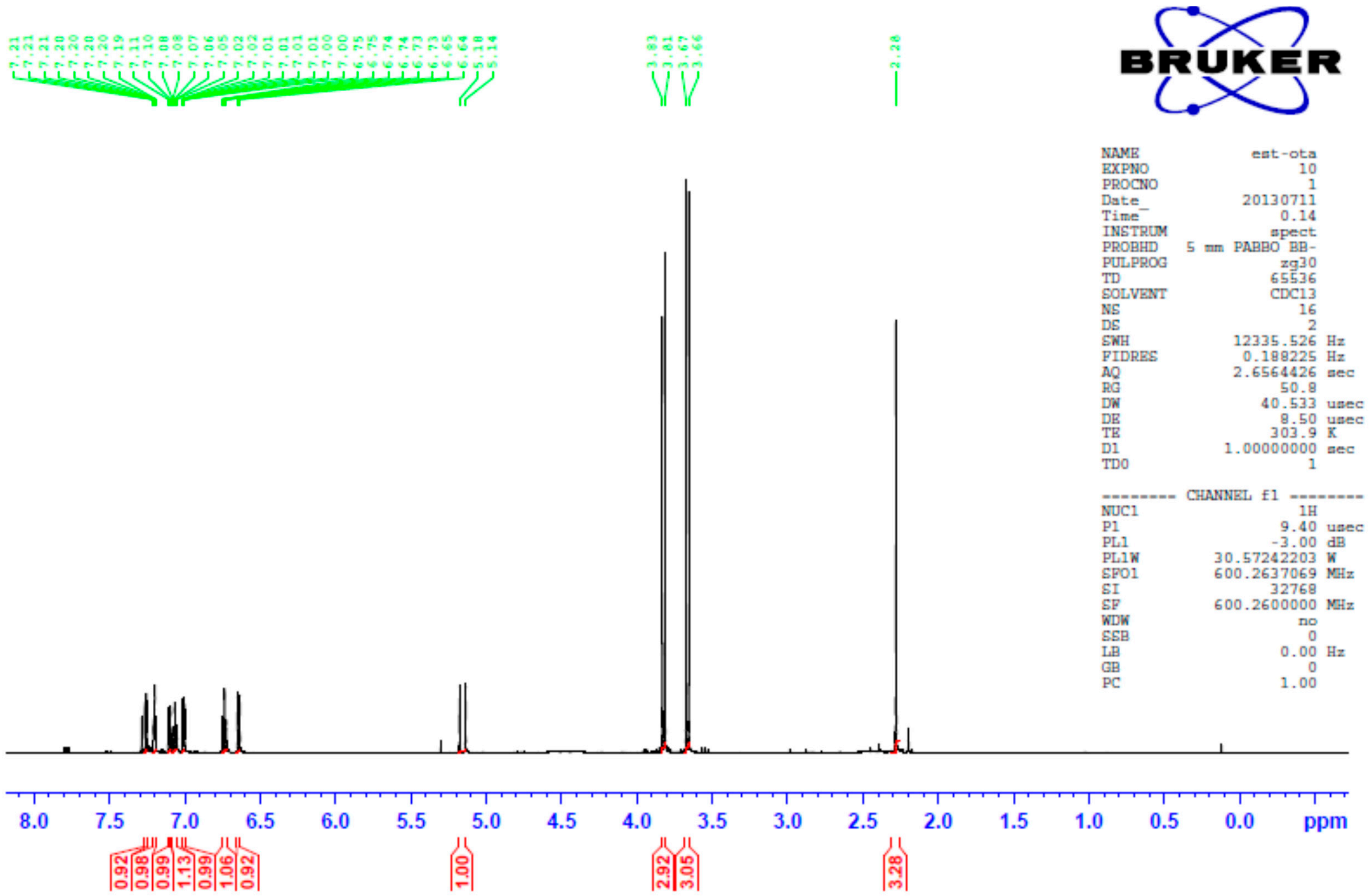

Figure S9. ^{1}H-NMR spectrum of dimethyl *N*-(2-methylphenyl)amino(2-thienyl)methylphosphonate (**2a**).

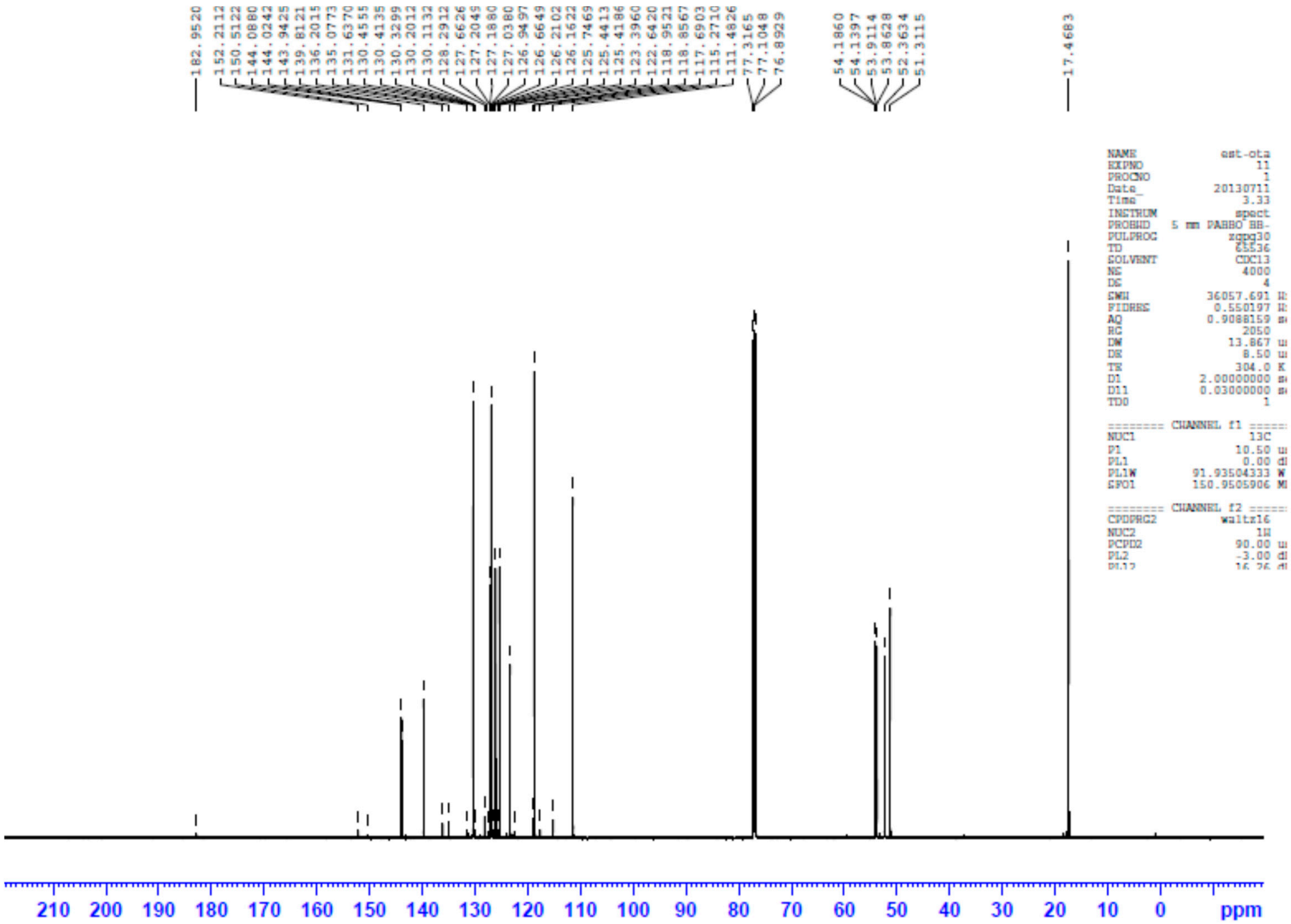

Figure S10. ^{13}C-NMR spectrum of dimethyl *N*-(2-methylphenyl)amino(2-thienyl)methylphosphonate (**2a**).

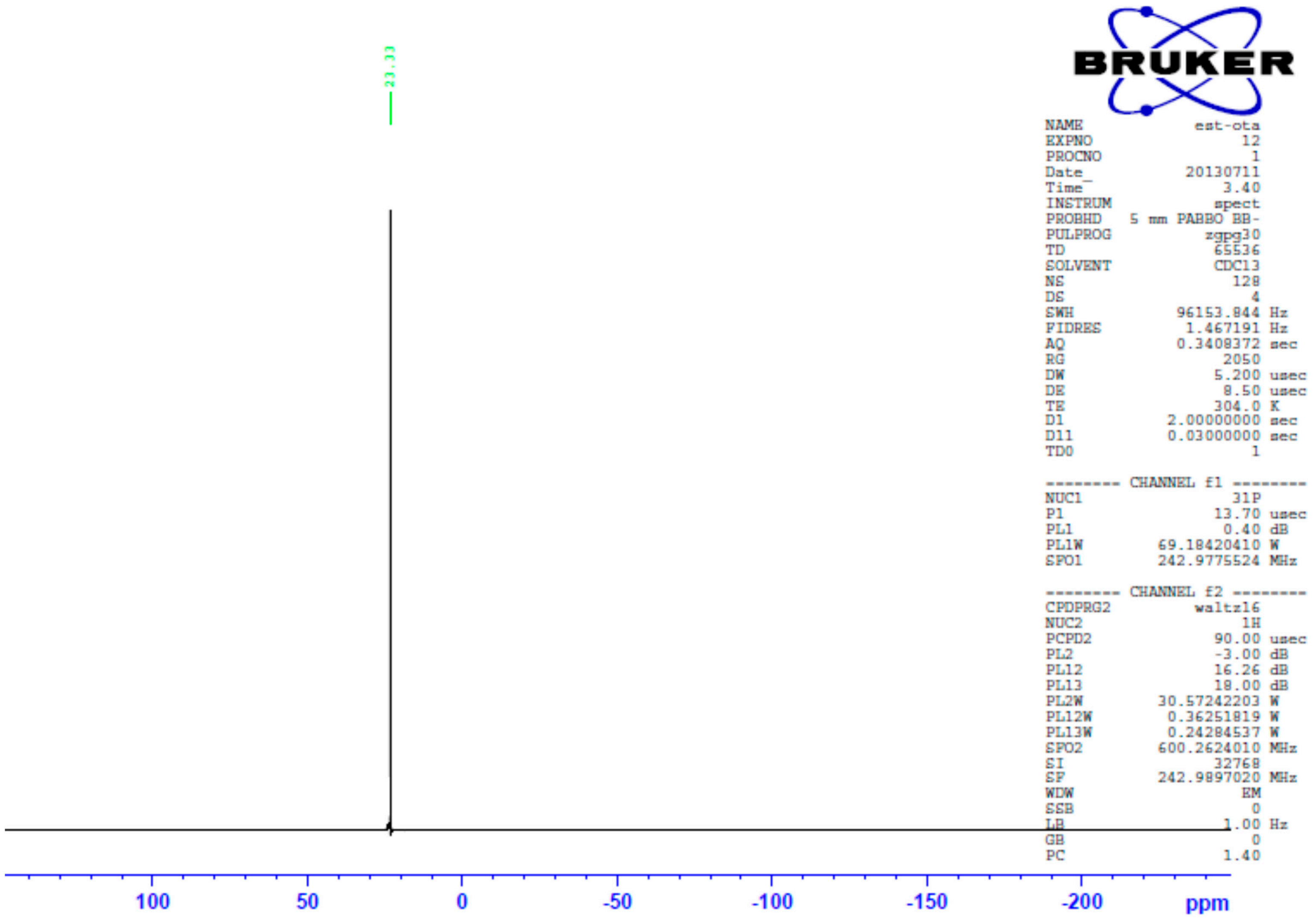

Figure S11. ^{31}P-NMR spectrum of dimethyl *N*-(2-methylphenyl)amino(2-thienyl)methylphosphonate (**2a**),

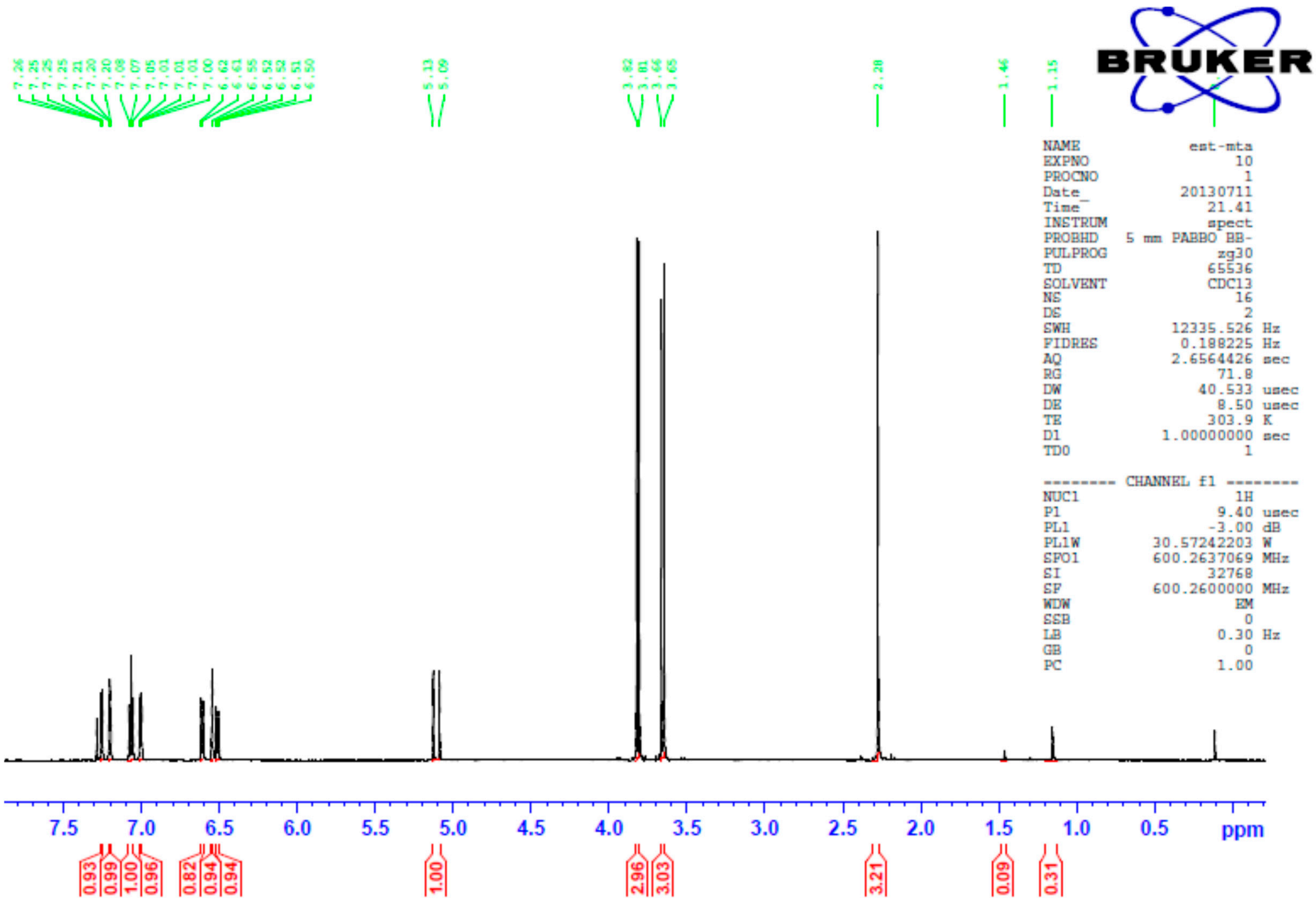

Figure S12. ^{1}H-NMR spectrum of dimethyl *N*-(3-methylphenyl)amino(2-thienyl)methylphosphonate (**2b**).

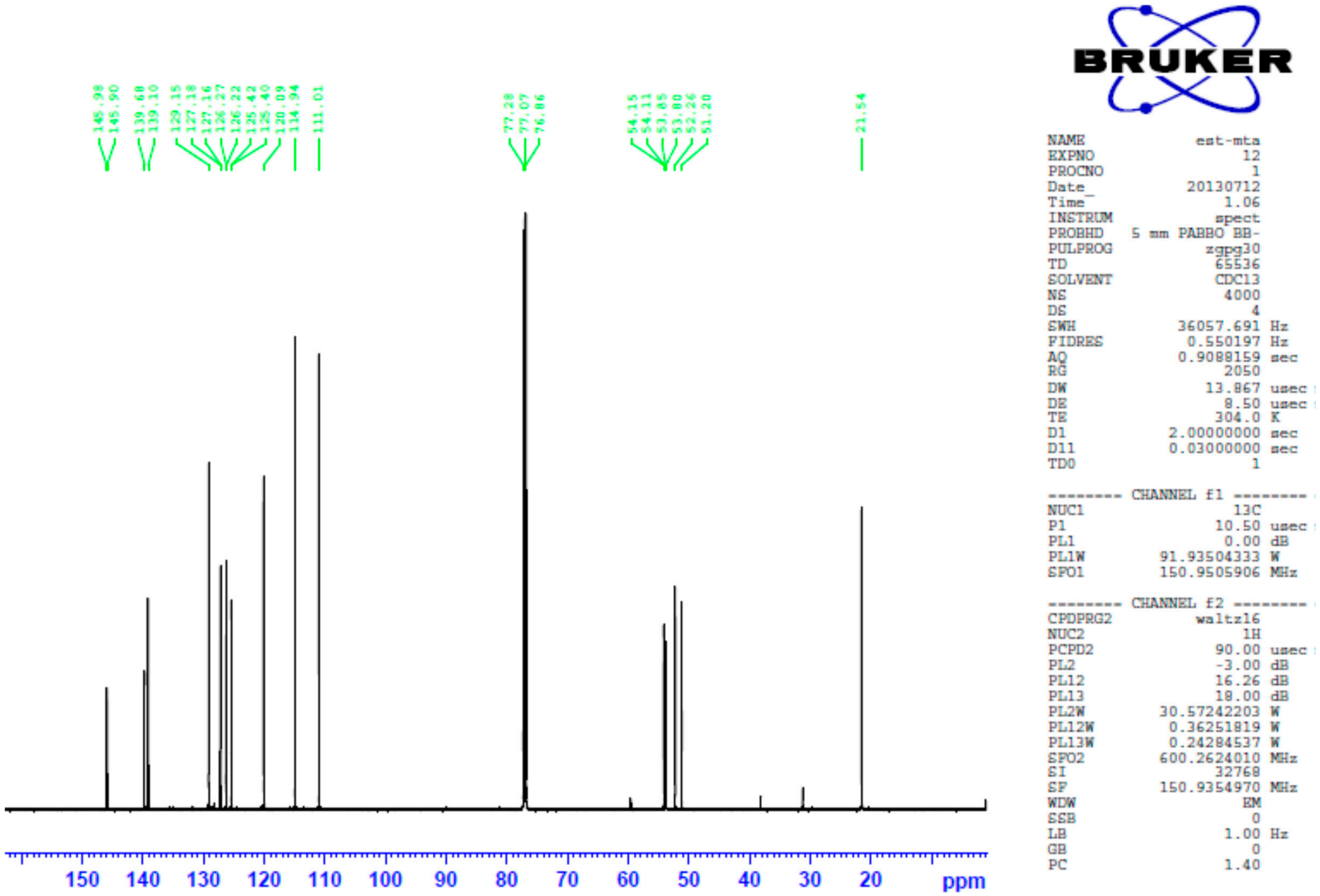

Figure S13. ^{13}C-NMR spectrum of dimethyl *N*-(3-methylphenyl)amino(2-thienyl)methylphosphonate (**2b**).

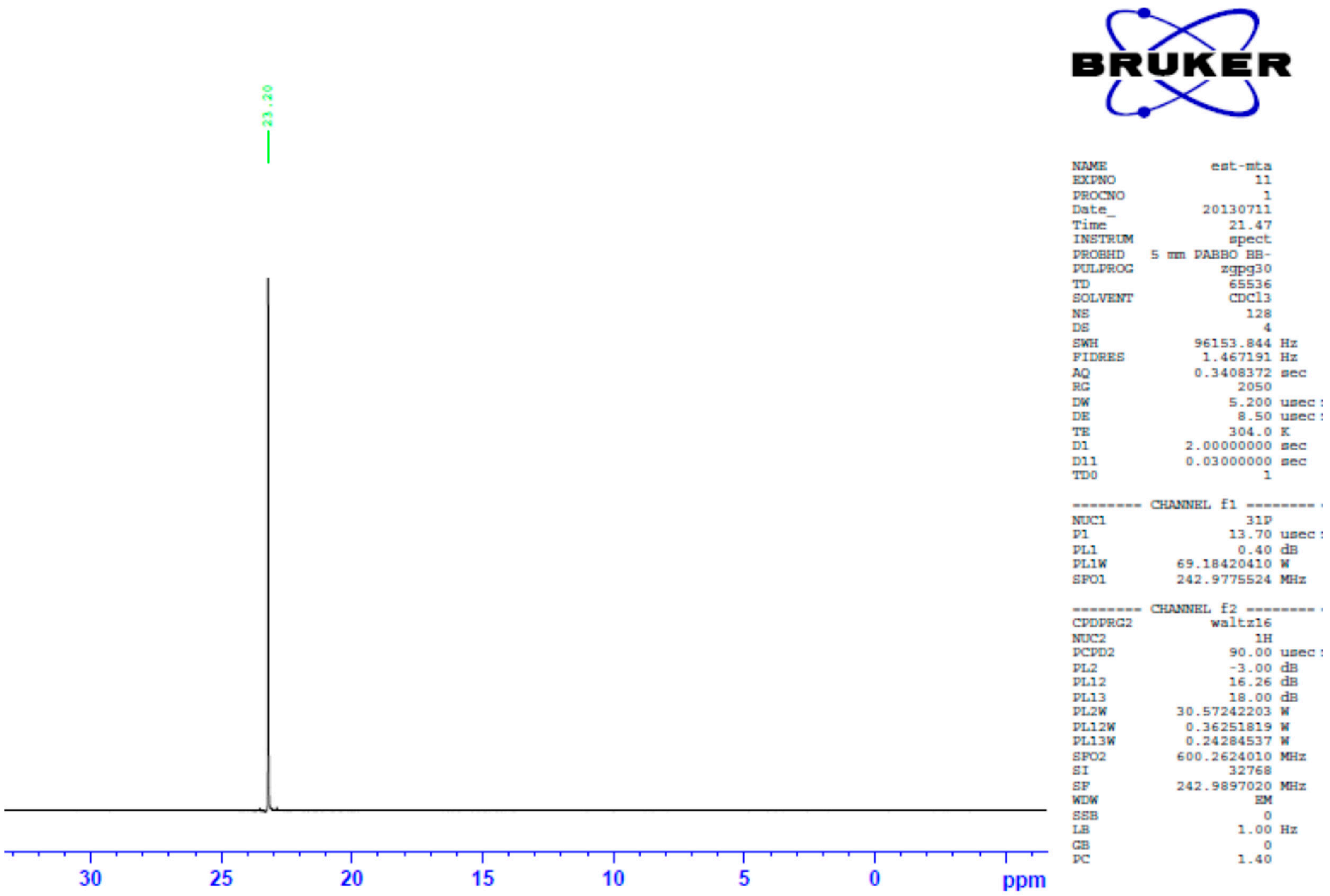

Figure S14. ^{31}P-NMR spectrum of dimethyl *N*-(3-methylphenyl)amino(2-thienyl)methylphosphonate (**2b**).

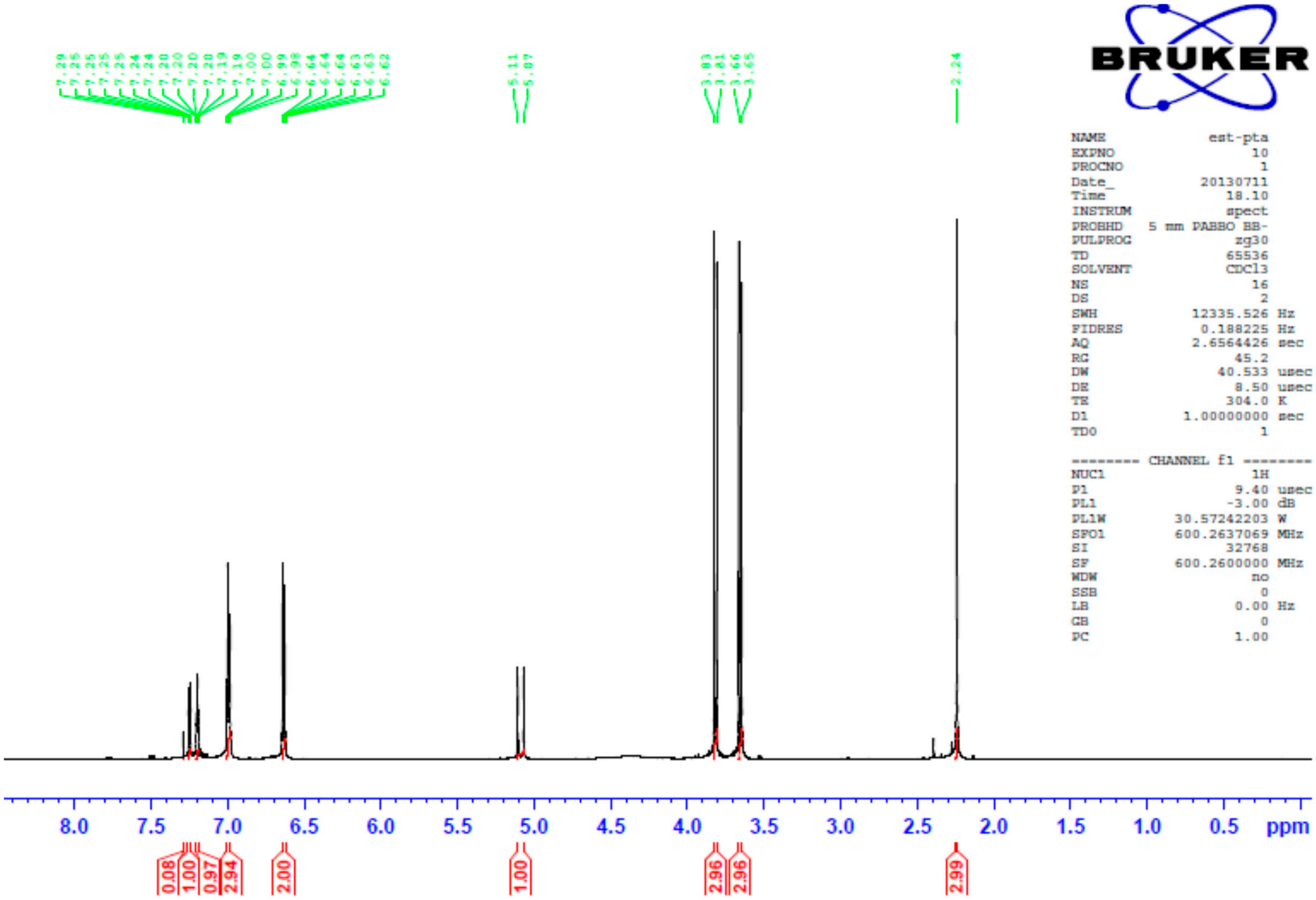

Figure S15. ^{1}H-NMR spectrum of dimethyl *N*-(4-methylphenyl)amino(2-thienyl)methylphosphonate (**2c**).

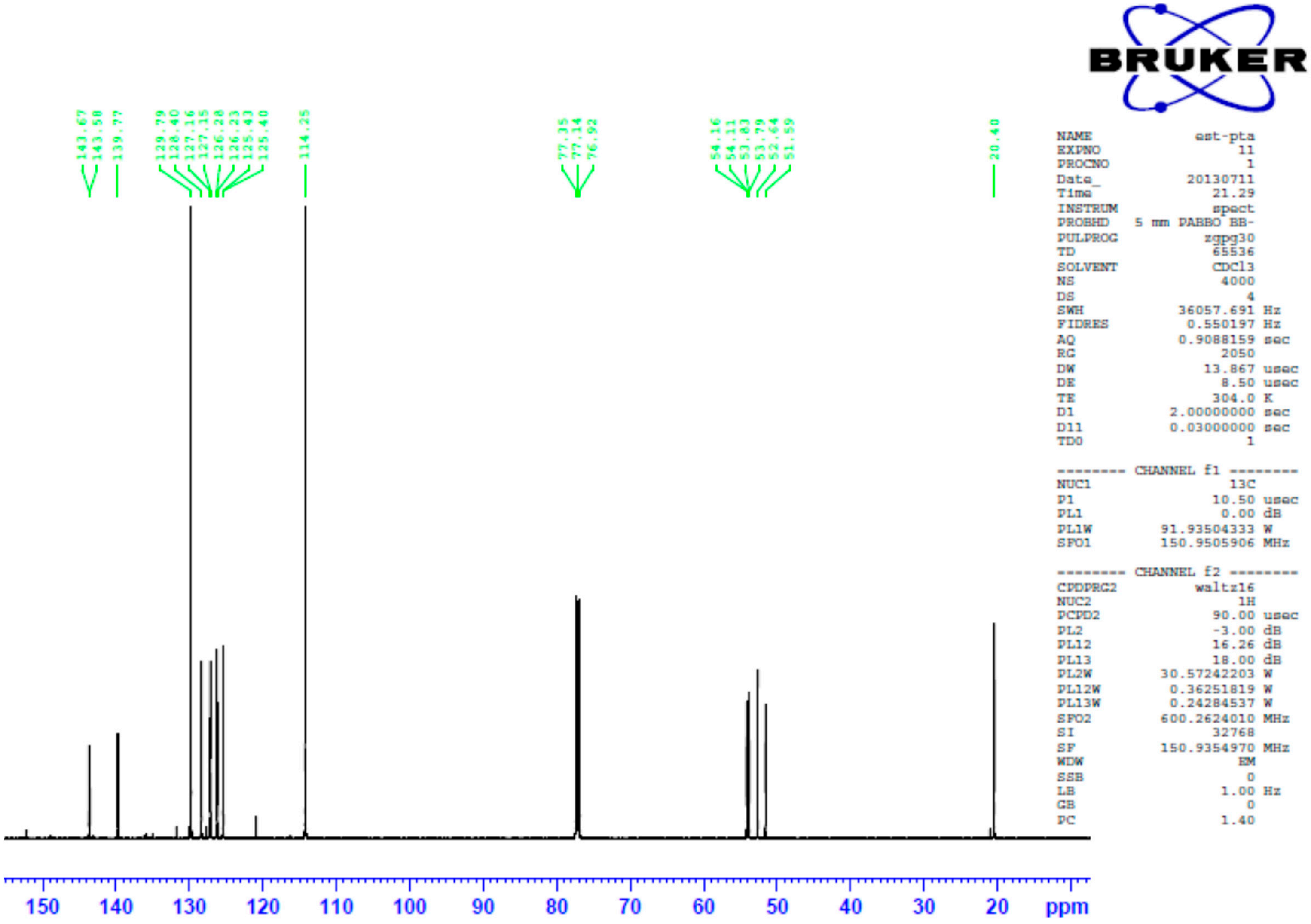

Figure S16. ^{13}C-NMR spectrum of dimethyl *N*-(4-methylphenyl)amino(2-thienyl)methylphosphonate (**2c**).

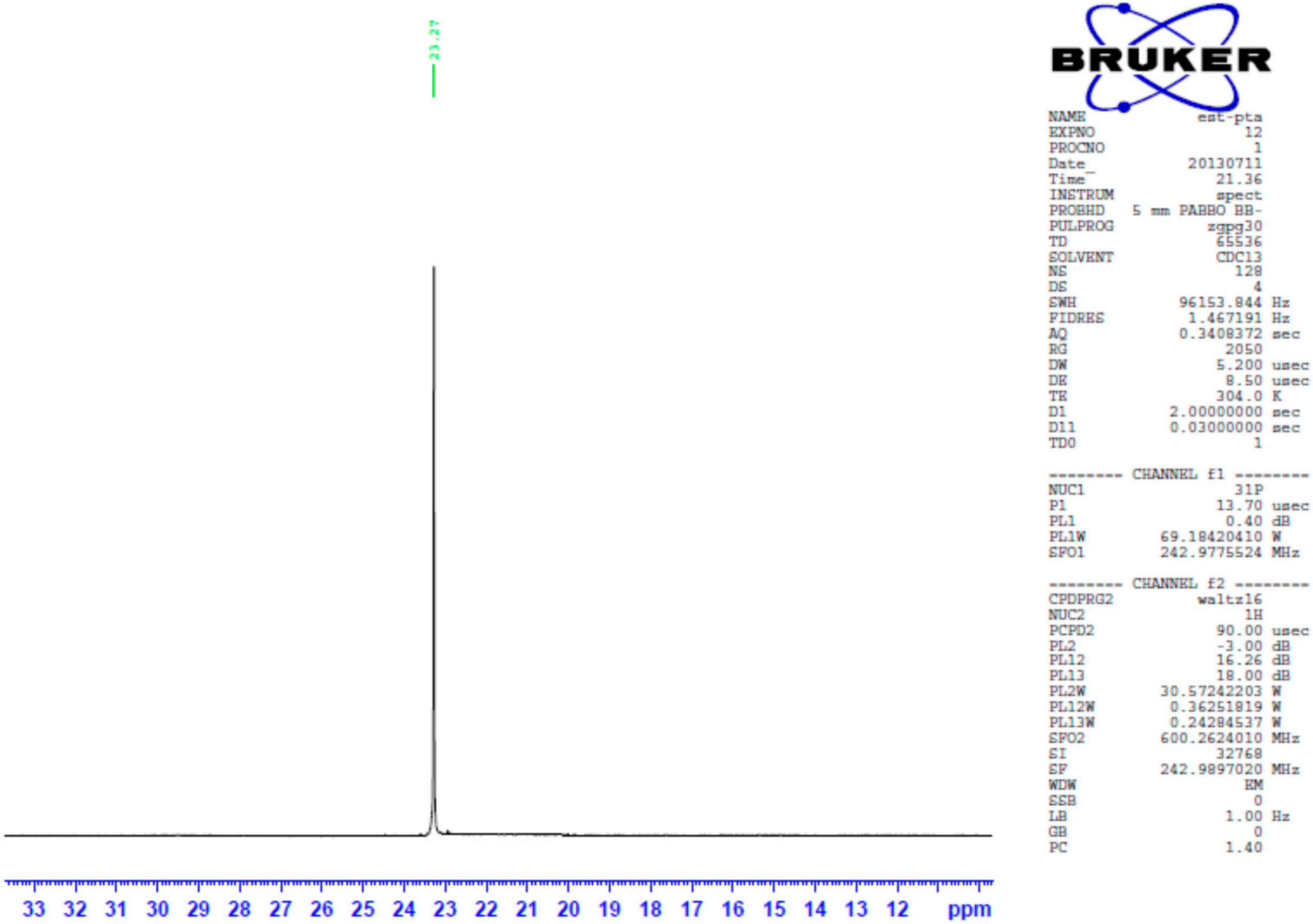

Figure S17. ^{31}P-NMR spectrum of dimethyl *N*-(4-methylphenyl)amino(2-thienyl)methylphosphonate (**2c**).

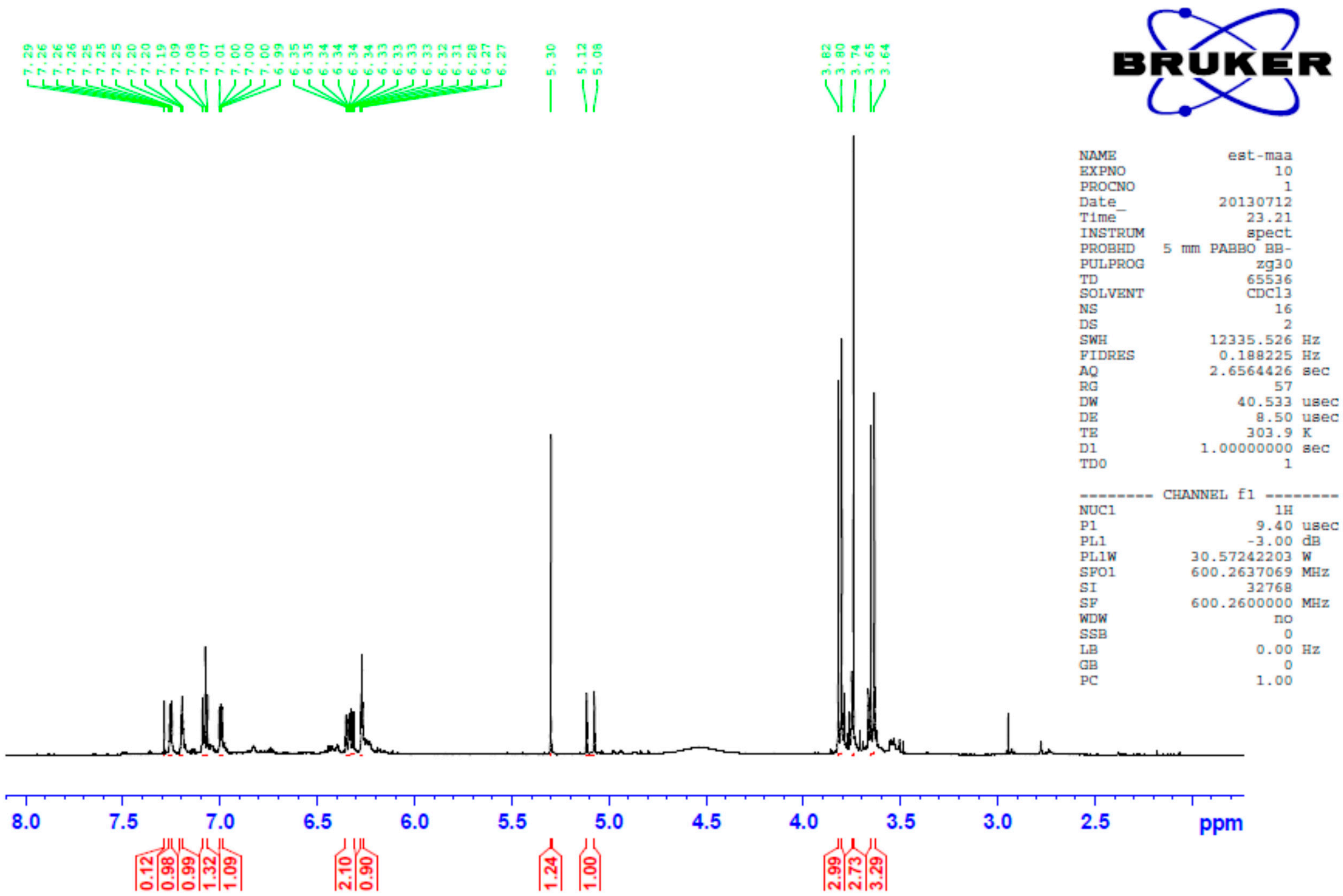

Figure S18. ^{1}H-NMR spectrum of dimethyl *N*-(3-methoxyphenyl)amino(2-thienyl)methylphosphonate (**2d**).

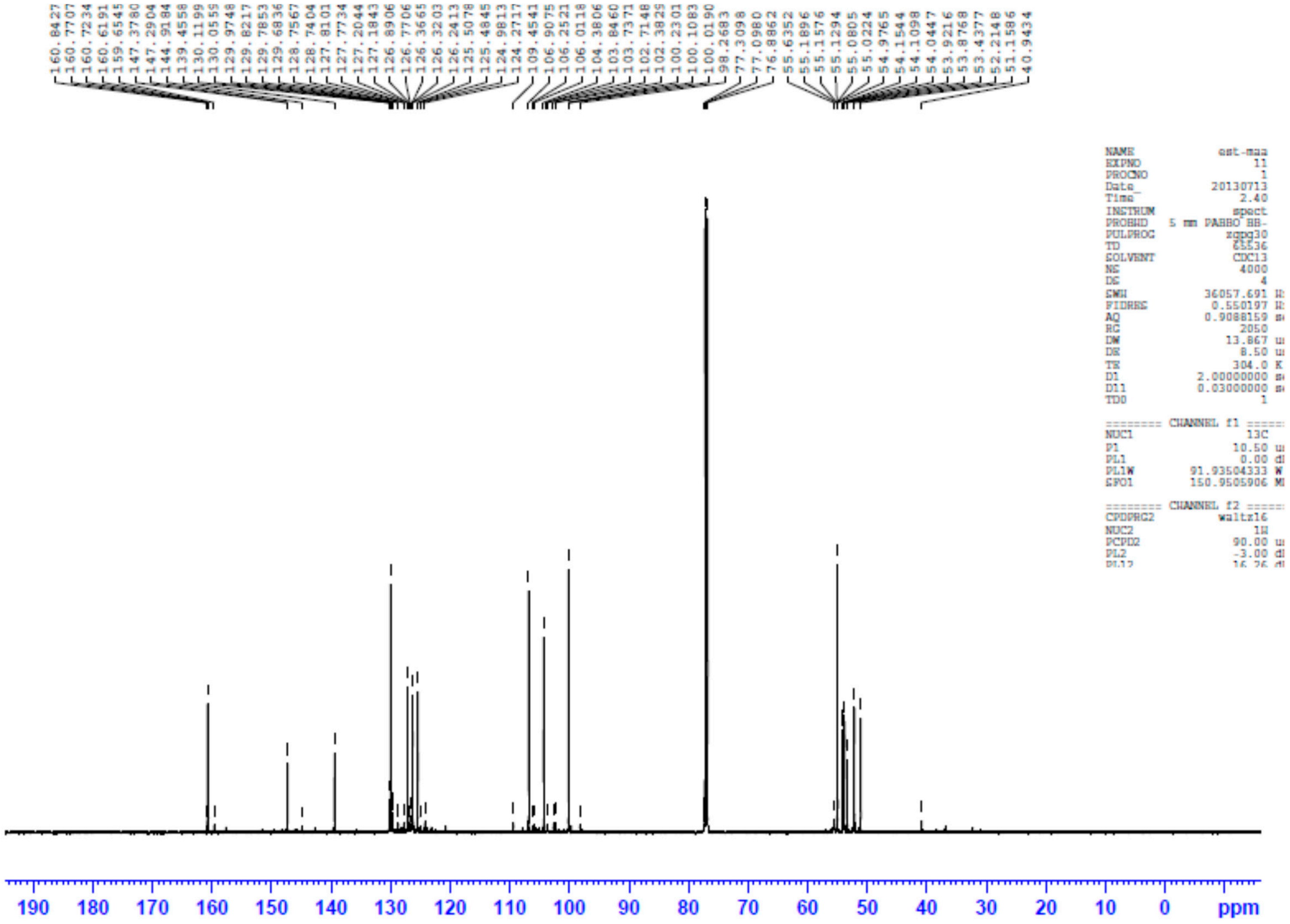

Figure S19. ^{13}C-NMR spectrum of dimethyl *N*-(3-methoxyphenyl)amino(2-thienyl)methylphosphonate (**2d**).

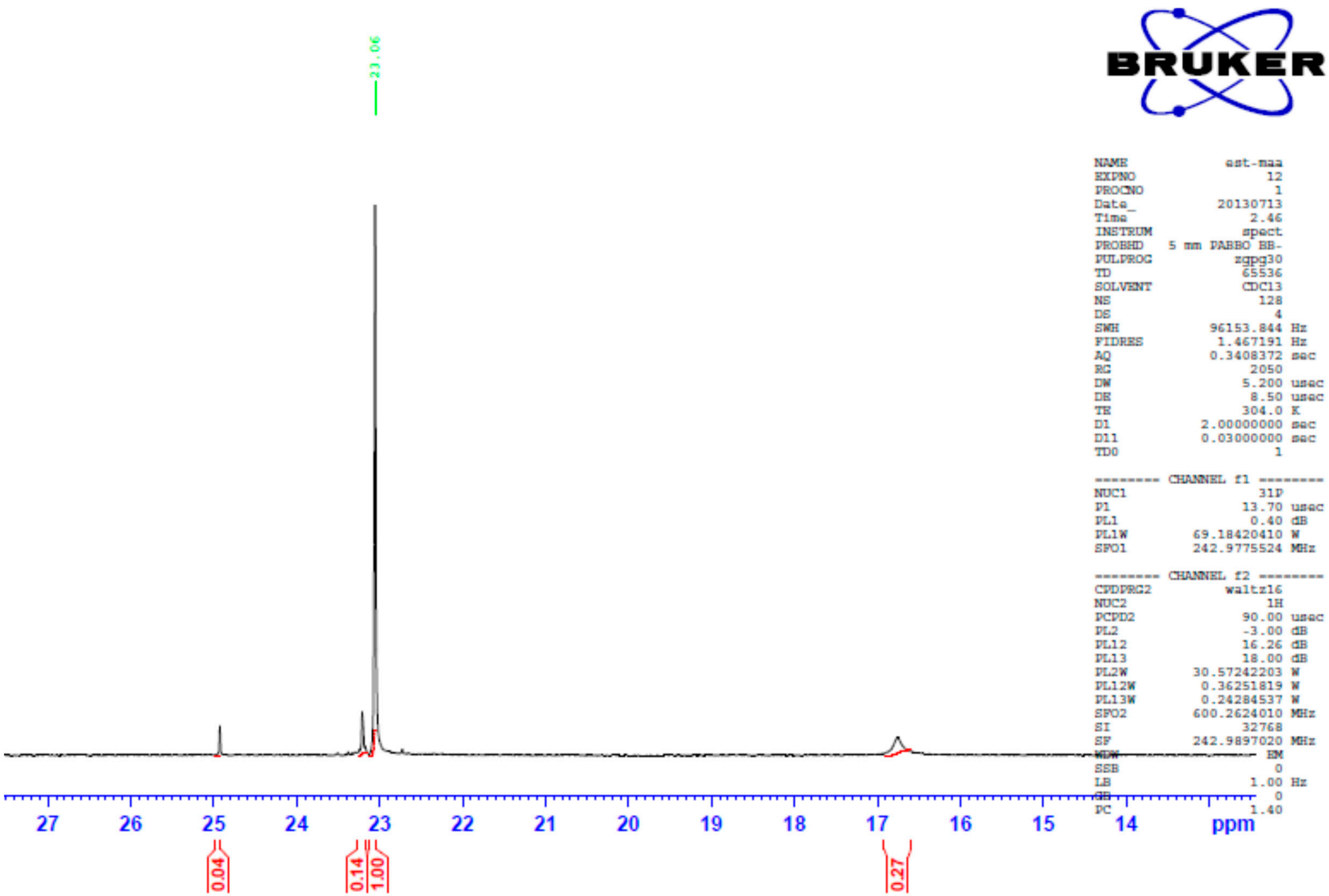

Figure S20. ^{31}P-NMR spectrum of dimethyl *N*-(3-methoxyphenyl)amino(2-thienyl)methylphosphonate (**2d**).

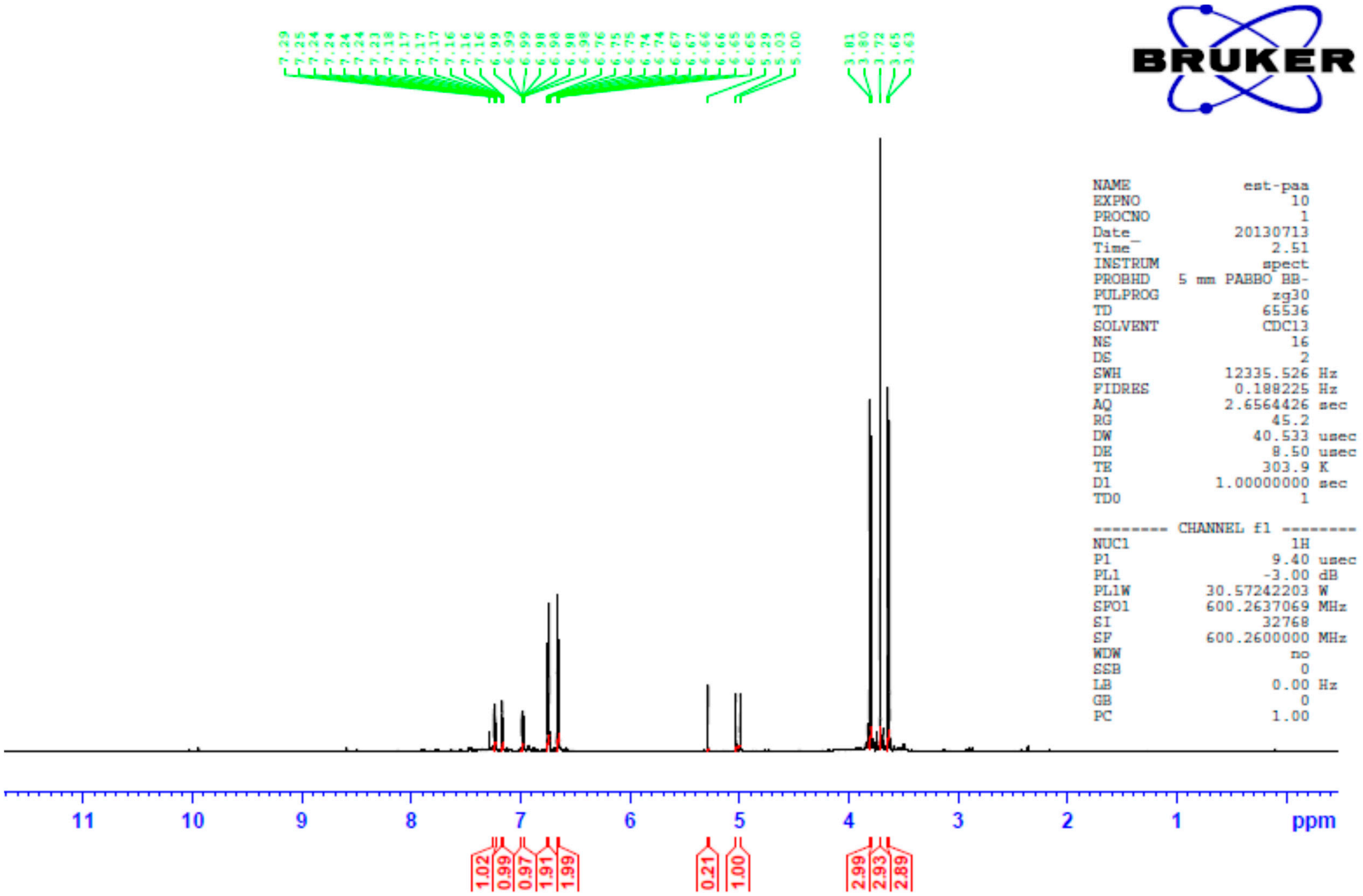

Figure S21. ^{1}H-NMR spectrum of dimethyl *N*-(4-methoxyphenyl)amino(2-thienyl)methylphosphonate (**2e**).

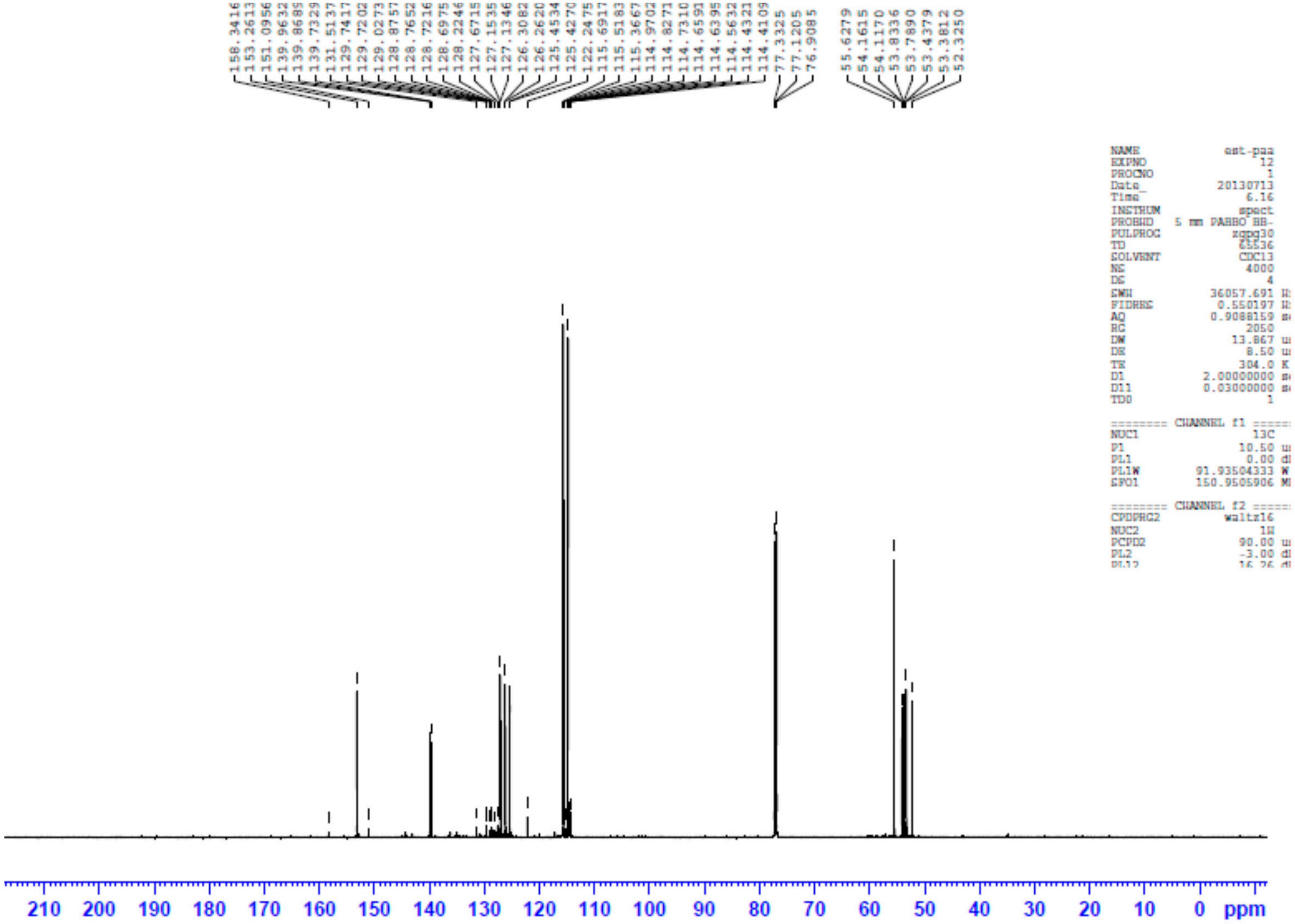

Figure S22. ^{13}C-NMR spectrum of dimethyl *N*-(4-methoxyphenyl)amino(2-thienyl)methylphosphonate **(2e).**

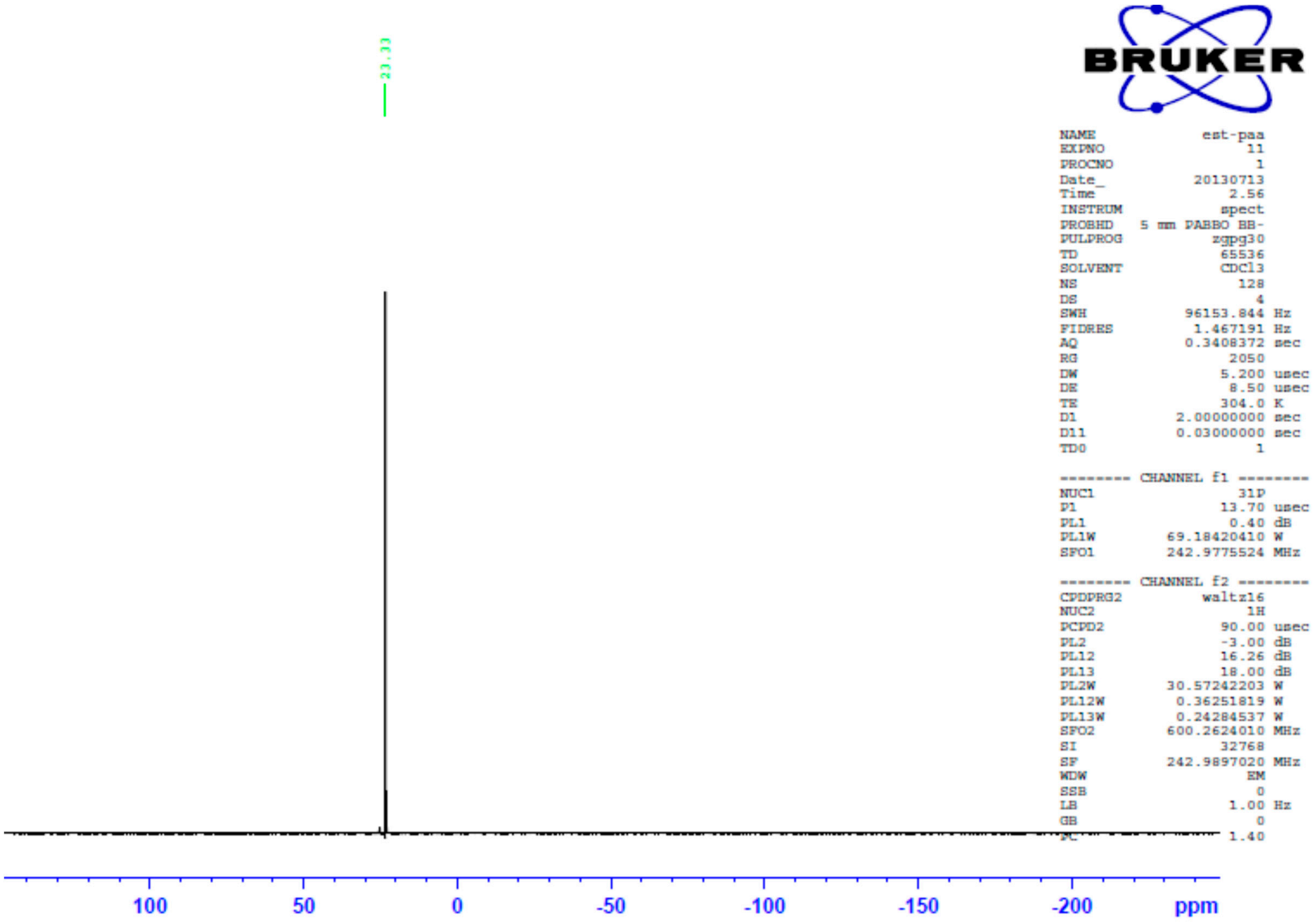

Figure S23. ^{31}P-NMR spectrum of dimethyl *N*-(4-methoxyphenyl)amino(2-thienyl)methylphosphonate (**2e**).

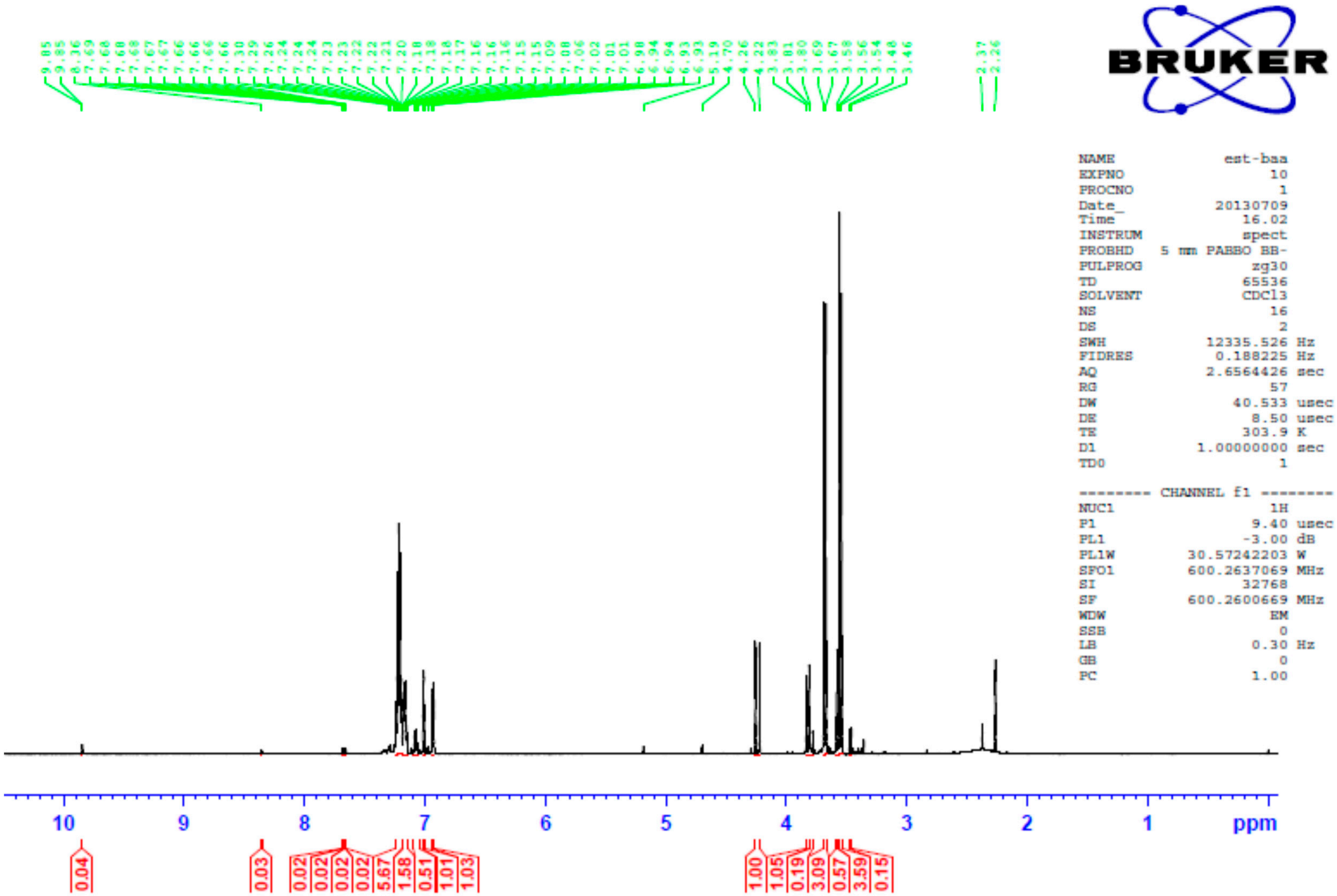

Figure S24. ^{1}H-NMR spectrum of dimethyl *N*-benzylamino(2-thienyl)methylphosphonate (**2f**).

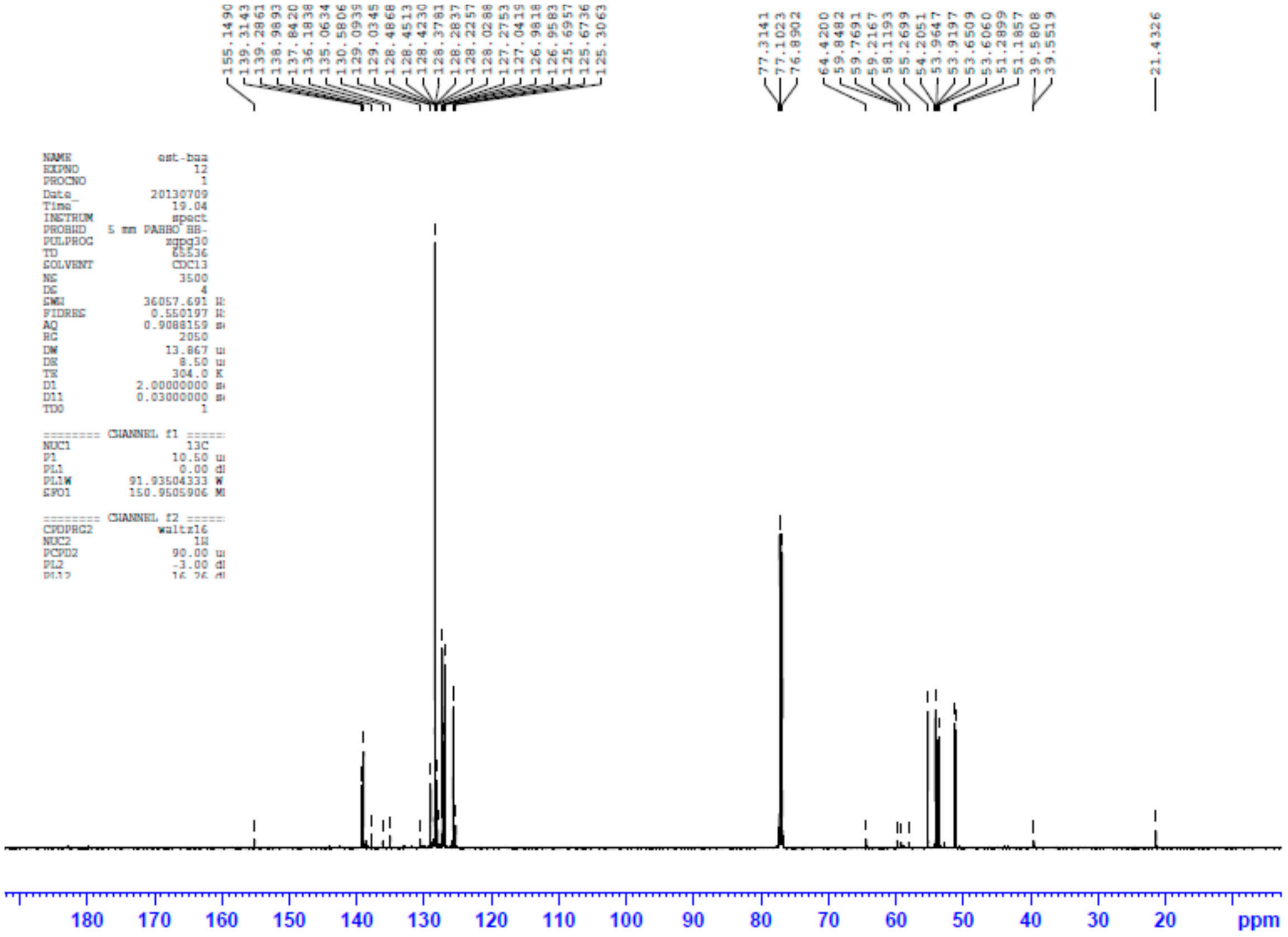

Figure S25. ^{13}C-NMR spectrum of dimethyl *N*-benzylamino(2-thienyl)methylphosphonate (**2f**).

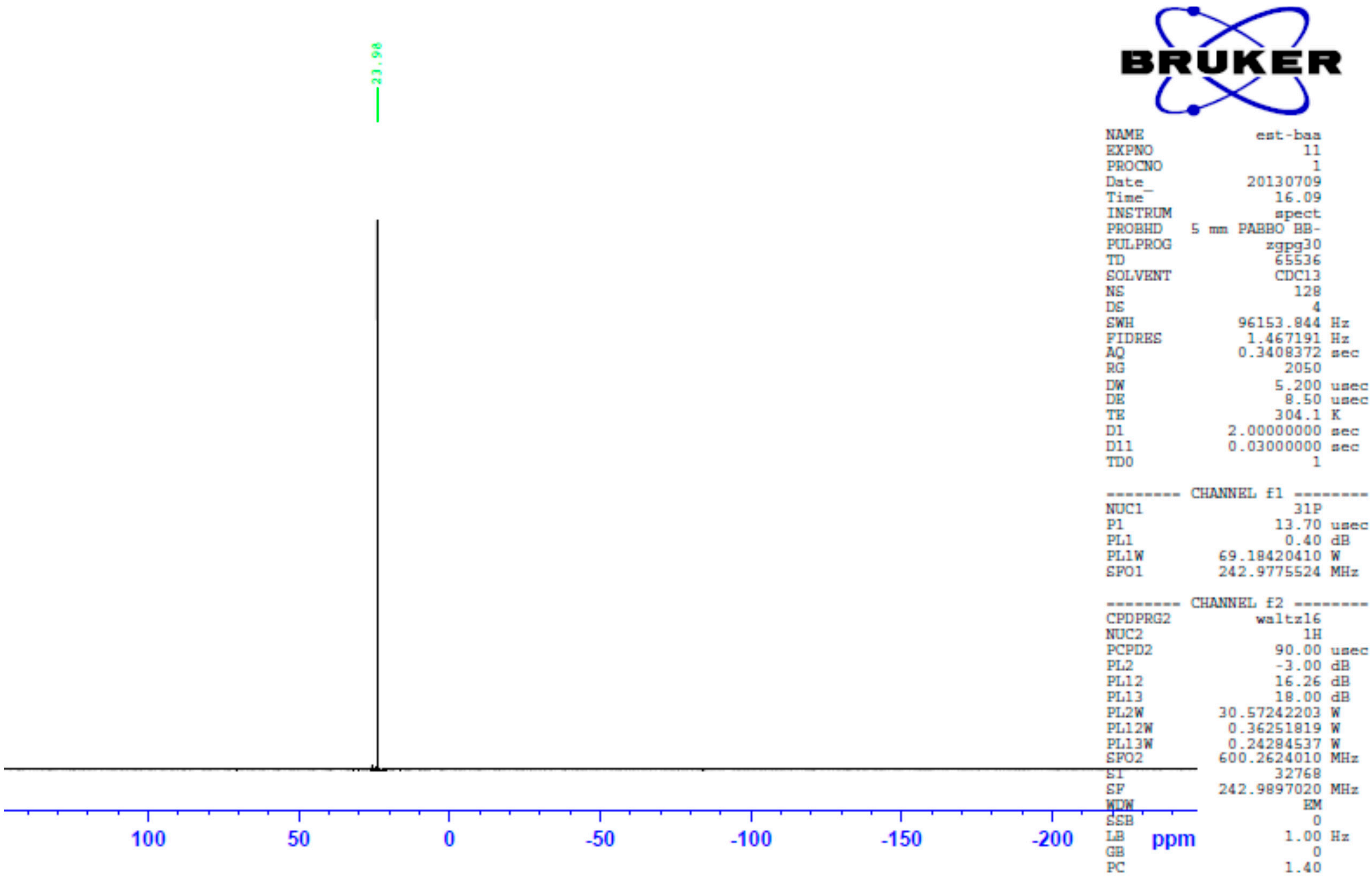

Figure S26. ^{31}P-NMR spectrum of dimethyl *N*-benzylamino(2-thienyl)methylphosphonate (**2f**).

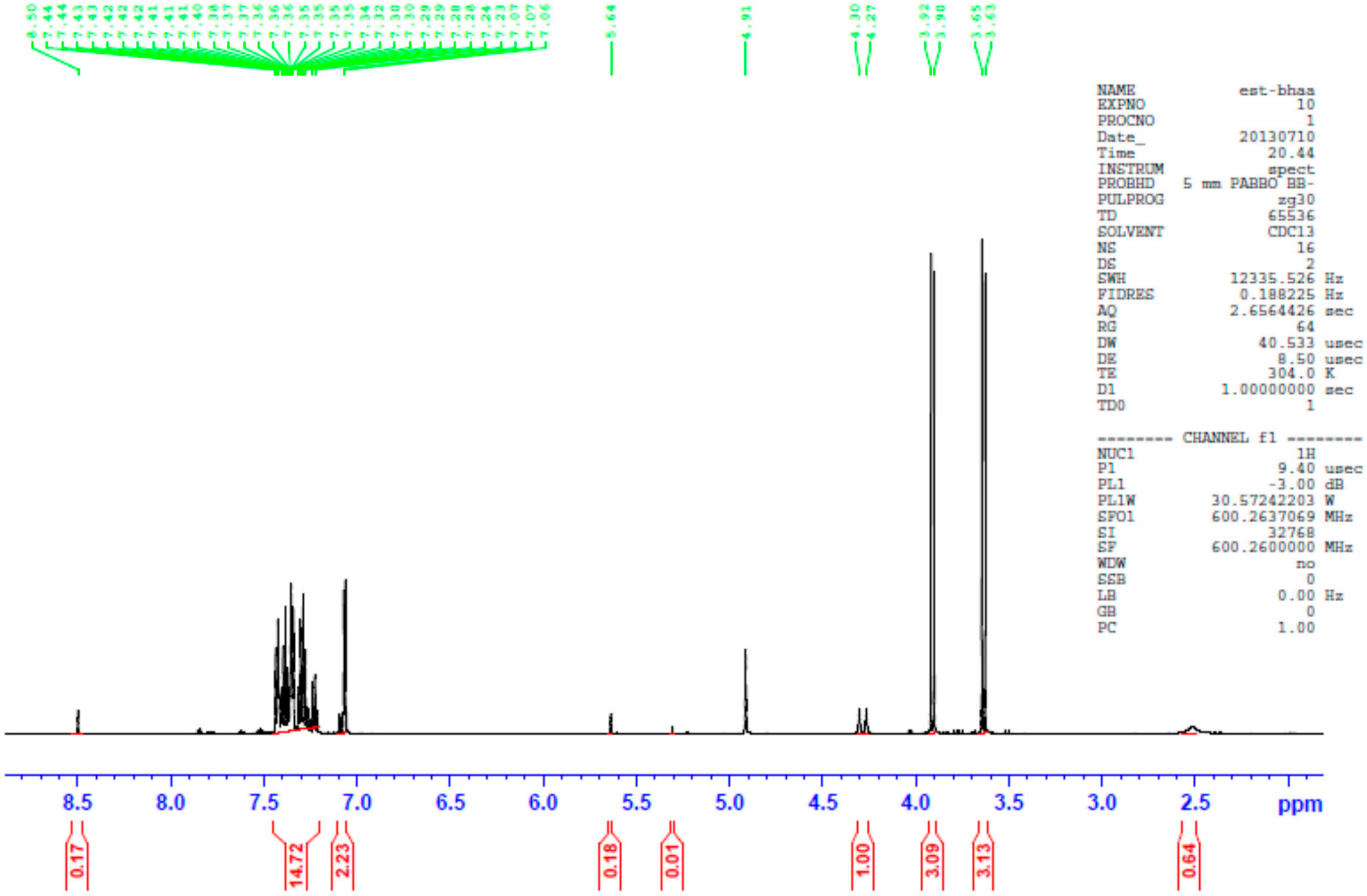

Figure S27. ^{1}H-NMR spectrum of dimethyl *N*-benzhydrylamino(2-thienyl)methylphosphonate (**2g**).

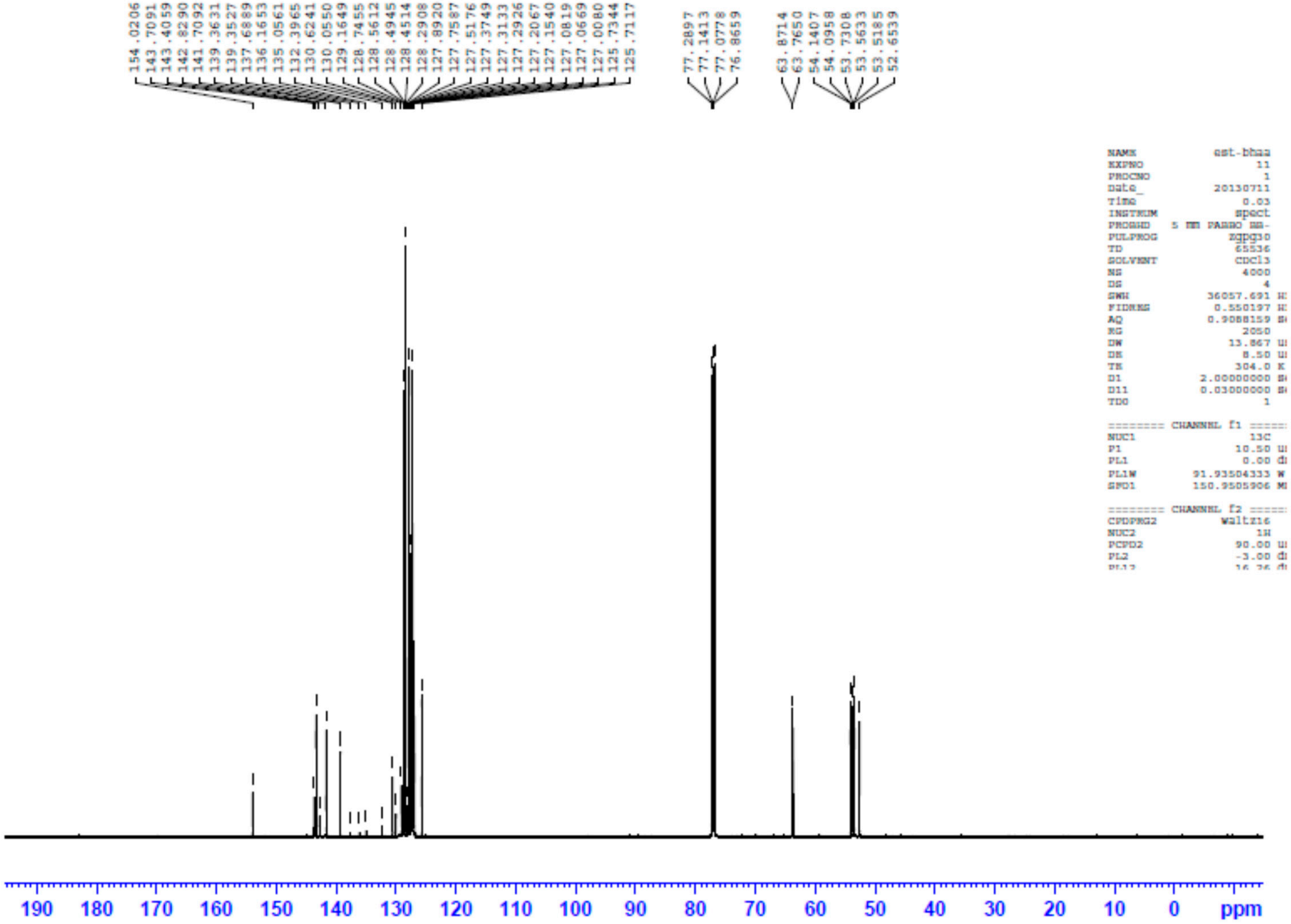

Figure S28. ^{13}C-NMR spectrum of dimethyl *N*-benzhydrylamino(2-thienyl)methylphosphonate (**2g**).

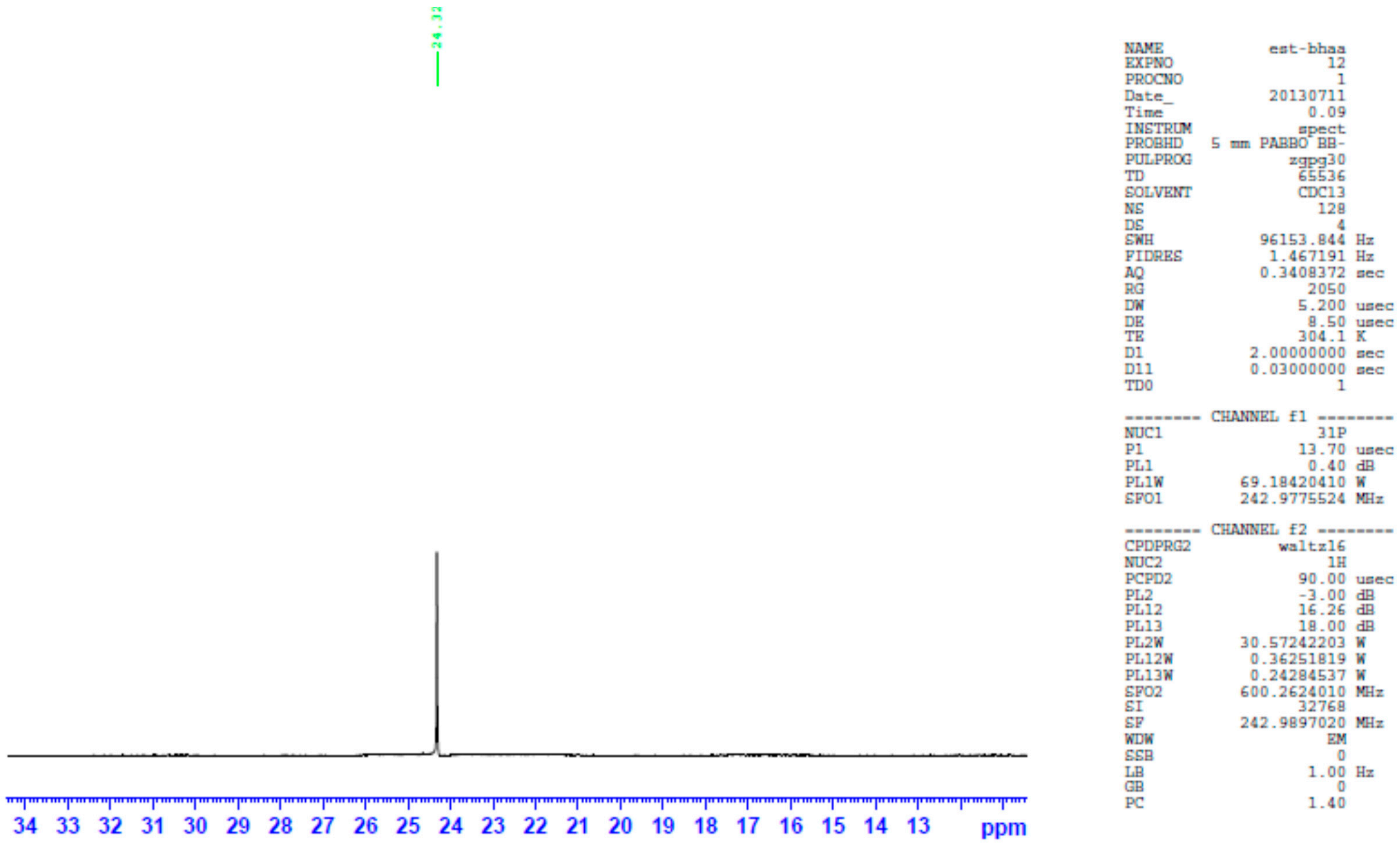

Figure S29. ^{31}P-NMR spectrum of dimethyl *N*-benzhydrylamino(2-thienyl)methylphosphonate (**2g**).

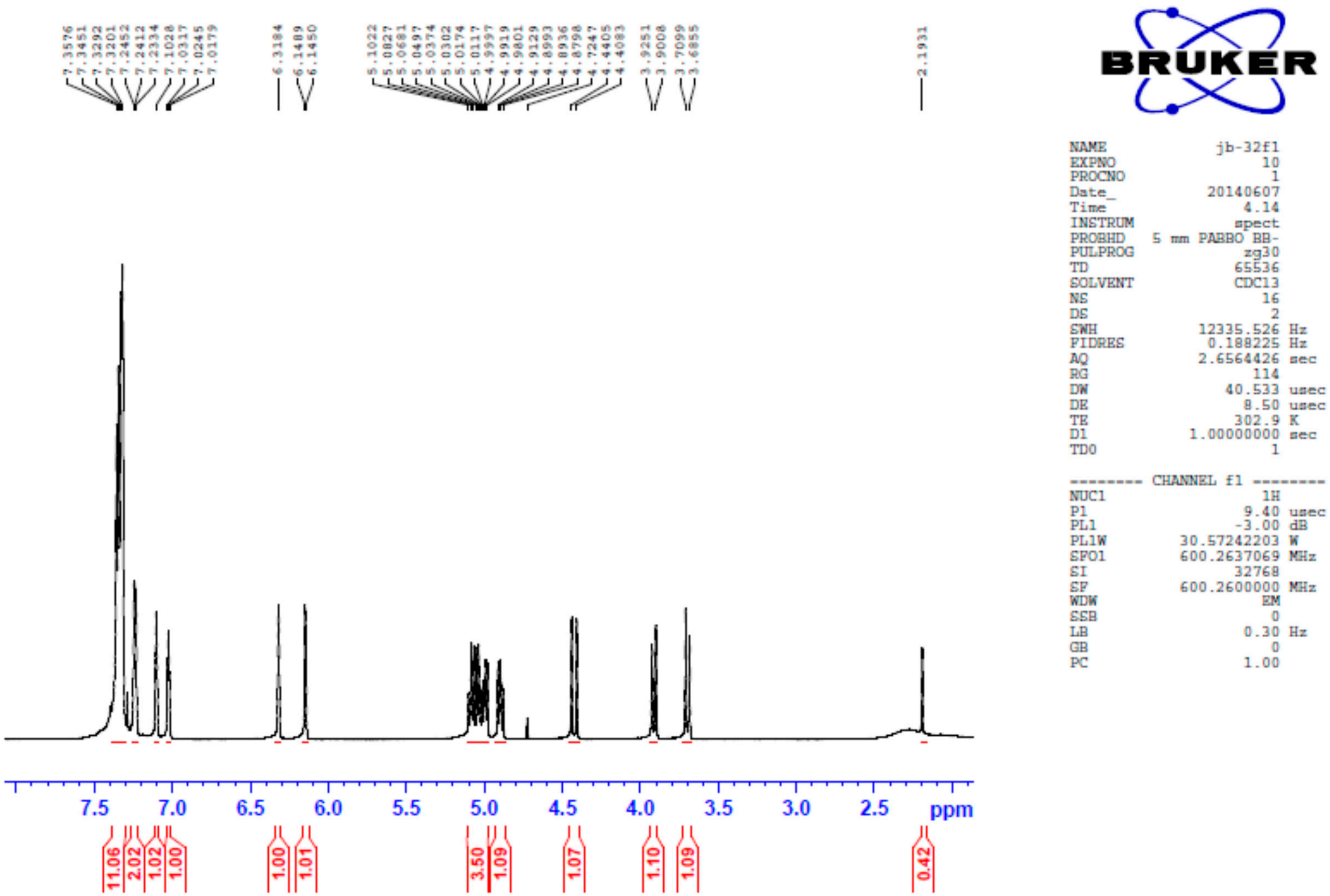

Figure S30. ^{1}H-NMR spectrum of dibenzyl *N*-furfurylamino(2-thienyl)methylphosphonate (**2h**).

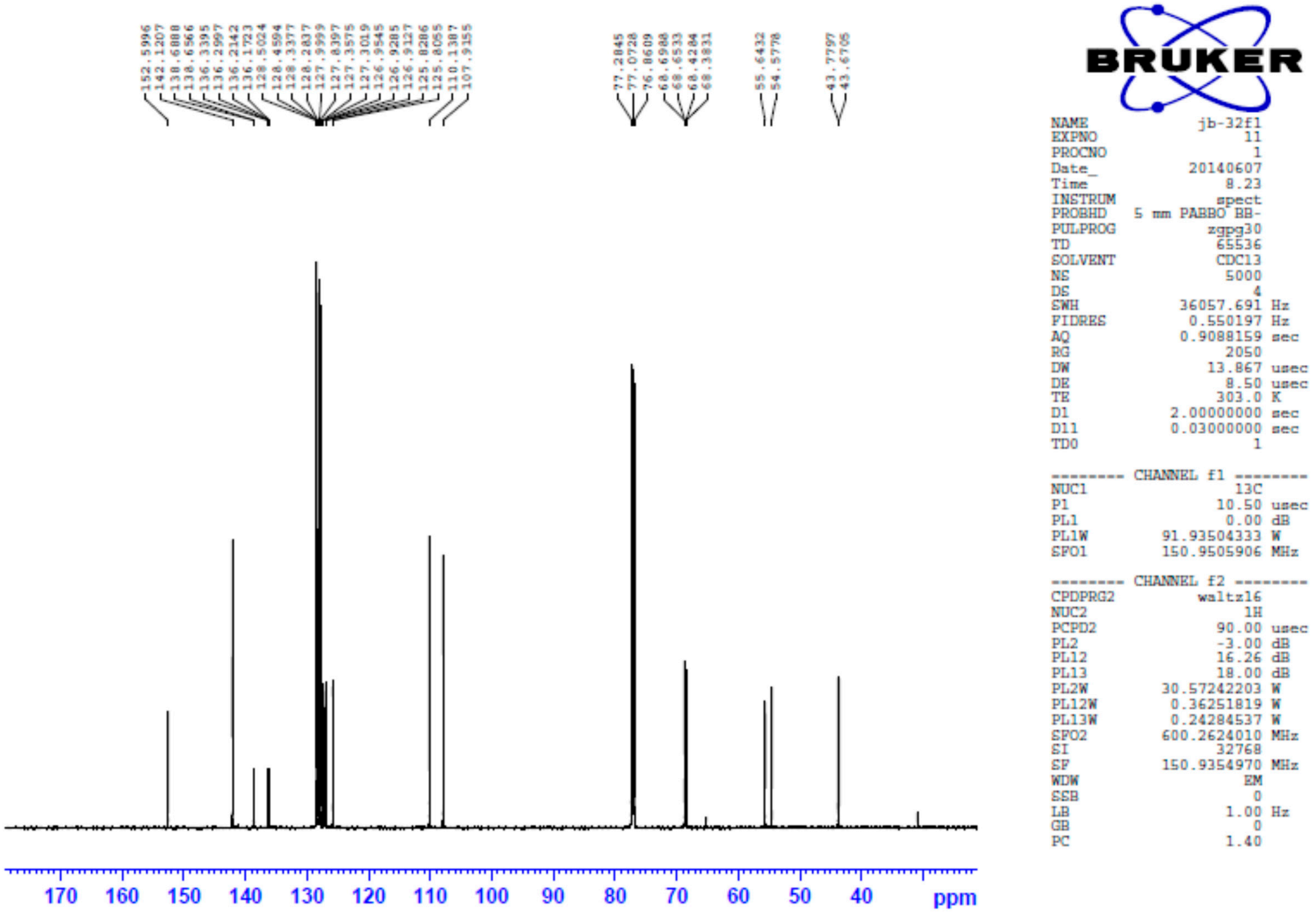

Figure S31. ^{13}C-NMR spectrum of dibenzyl *N*-furfurylamino(2-thienyl)methylphosphonate (**2h**).

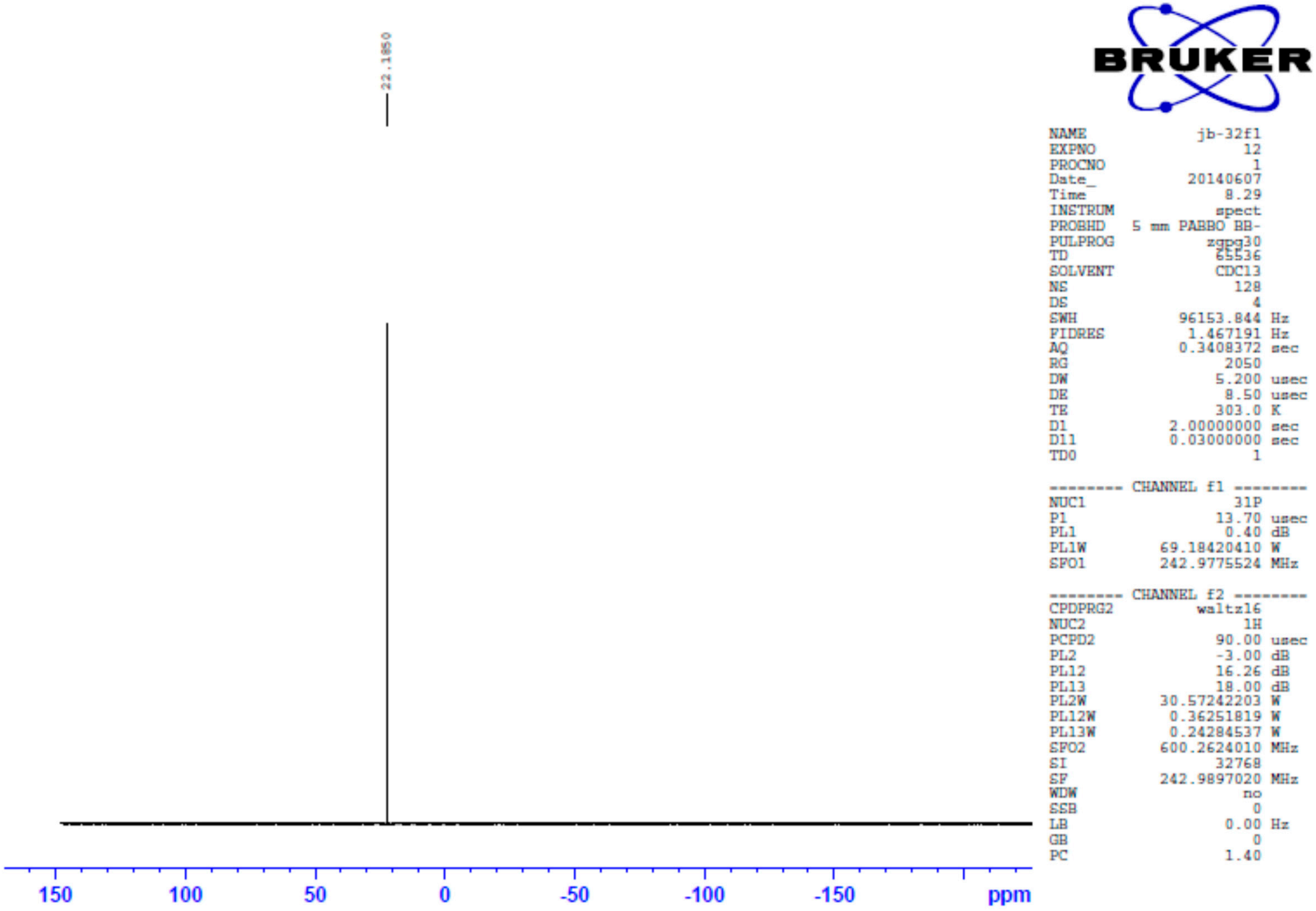

Figure S32. ^{31}P-NMR spectrum of dibenzyl *N*-furfurylamino(2-thienyl)methylphosphonate (**2h**).
